# Herb and Spices in Colorectal Cancer Prevention and Treatment: A Narrative Review

**DOI:** 10.3389/fphar.2022.865801

**Published:** 2022-06-30

**Authors:** Md. Sanower Hossain, Md. Abdul Kader, Khang Wen Goh, Maidul Islam, Md. Sharif Khan, Md. Harun-Ar Rashid, Der Jiun Ooi, Henrique Douglas Melo Coutinho, Yaser Mohammed Al-Worafi, Said Moshawih, Ya Chee Lim, K. M. Kaderi Kibria, Long Chiau Ming

**Affiliations:** ^1^ Department of Biomedical Science, Kulliyyah of Allied Health Sciences, International Islamic University Malaysia, Kuantan, Malaysia; ^2^ Faculty of Science, Sristy College of Tangail, Tangail, Bangladesh; ^3^ Department of Biotechnology and Genetic Engineering, Mawlana Bhashani Science and Technology University, Tangail, Bangladesh; ^4^ Faculty of Data Science and Information Technology, INTI International University, Nilai, Malaysia; ^5^ Digital Medical Systems Ltd., Dhaka, Bangladesh; ^6^ Department of Nutrition and Food Engineering, Faculty of Allied Health Sciences, Daffodil International University, Dhaka, Bangladesh; ^7^ Department of Oral Biology & Biomedical Sciences, Faculty of Dentistry, MAHSA University, Jenjarom, Malaysia; ^8^ Departamento de Química Biológica, Laboratório de Microbiologia E Biologia Molecular—LMBM, Universidade Regional Do Cariri, URCA, Crato, Brazil; ^9^ College of Medical Sciences, Azal University for Human Development, Amran, Yemen; ^10^ College of Pharmacy, University of Science and Technology of Fujairah, Fujairah, United Arab Emirates; ^11^ PAP Rashidah Sa’adatul Bolkiah Institute of Health Sciences, Universiti Brunei Darussalam, Bandar Seri Begawan, Brunei

**Keywords:** biomolecules, colon cancer, drug resistance, functional foods, management, nutraceuticals, prevalence

## Abstract

Colorectal cancer (CRC) is the second most deadly cancer worldwide. CRC management is challenging due to late detection, high recurrence rate, and multi-drug resistance. Herbs and spices used in cooking, practised for generations, have been shown to contain CRC protective effect or even be useful as an anti-CRC adjuvant therapy when used in high doses. Herbs and spices contain many bioactive compounds and possess many beneficial health effects. The chemopreventive properties of these herbs and spices are mainly mediated by the BCL-2, K-ras, and MMP pathways, caspase activation, the extrinsic apoptotic pathway, and the regulation of ER-stress-induced apoptosis. As a safer natural alternative, these herbs and spices could be good candidates for chemopreventive or chemotherapeutic agents for CRC management because of their antiproliferative action on colorectal carcinoma cells and inhibitory activity on angiogenesis. Therefore, in this narrative review, six different spices and herbs: ginger (*Zingiber officinale* Roscoe), turmeric (*Curcuma longa* L.), garlic (*Allium sativum* L.), fenugreek (*Trigonella foenum-graecum* L.), sesame (*Sesamum indicum* L.), and flaxseed (*Linum usitatissimum* L.) used in daily cuisine were selected for this study and analyzed for their chemoprotective or chemotherapeutic roles in CRC management with underlying molecular mechanisms of actions. Initially, this study comprehensively discussed the molecular basis of CRC development, followed by culinary and traditional uses, current scientific research, and publications of selected herbs and spices on cancers. Lead compounds have been discussed comprehensively for each herb and spice, including anti-CRC phytoconstituents, antioxidant activities, anti-inflammatory properties, and finally, anti-CRC effects with treatment mechanisms. Future possible works have been suggested where applicable.

## 1 Introduction

### 1.1 Colorectal Cancer

Cancer is a leading cause of death that significantly affects the life expectancy of every nation. Colorectal cancer (CRC) is the third most commonly diagnosed and second most deadly cancer globally (WHO, 2021) that accounting for approximately 9.39% of death of all recorded cancers in 2020 (Ferlay et al., 2020; Hossain et al., 2022). CRC incidence is expected to double by 2035 worldwide due to the fast acceleration of diagnosed cases in the elderly. Less developed countries are expected to rise in diagnosed cases of CRC (Papamichael et al., 2015; Hossain et al., 2022).

The term CRC is specific to the large intestine and rectum, where it develops from the abnormal growth of glandular epithelial cells. This development occurs when epithelial cells acquire a succession of genetic or epigenetic alterations that provide them with a selective advantage of hyper-proliferation (Testa et al., 2018). These out of control growing cells produce a benign adenoma, which then progresses to carcinoma and metastasis by three major pathways: microsatellite instability (MSI), chromosomal instability (CIN), and CpG island methylator phenotype (CIMP) (Vogelstein et al., 1988; Nguyen and Duong, 2018; Malki et al., 2020). Like any other tumour or cancer, CRC is classified into stages: Stage 0 (carcinoma *in situ*) to stage IV. Standard treatment options for the stages 0 –II CRC are surgery, whereas stage III requires surgery and adjuvant chemotherapy, and stage IV and recurrence CRC involve surgery, chemotherapy, and targeted therapy (PDQ, 2021).

### 1.2 Colorectal Cancer Treatment Opportunity and Challenges

Regular screening can prevent CRC. As a polyp takes 10–15 years to be cancerous, detecting and removing polyps at an early stage is critical. However, only 40% of CRC are found at early stages, and sometimes CRC recurs after treatment (ACS, 2020). For CRC treatment strategy, Food and Drug Administration (FDA) approved at least 30 different drugs (Supplementary Table S1), and either singly or in combination with other drugs (Supplementary Table S2) are used for CRC treatment. These chemotherapeutic drugs are exposed to the cancer cells and simultaneously damage healthy cells. Consequently, these drugs manifest several adverse effects, including fatigue, headache, muscle pain, stomach pain, diarrhoea and vomiting, sore throat, blood abnormalities, constipation, neuronal damage, skin changes, memory problems, loss of appetite, and hair loss (ACS, 2020).

Even though the overall survival of individuals with advanced CRC has increased in recent decades due to new chemotherapy regimens (Supplementary Tables S1, S2); however, in nearly all patients with CRC, current systemic chemotherapies developed resistance (Cho and Hu, 2020), limiting the therapeutic efficacy of anti-cancer medicines and ultimately leading to chemotherapy failure. Chemotherapeutic drug resistance is a major issue in CRC treatment in the current clinical practice. Apart from this limitation, the access to diagnosis and treatment of CRC for survival is less accessible in developing countries, particularly for rural people, where about 44% of the world’s people currently live (World Bank, 2021). Therefore, nearly half the world’s population lacks the means to diagnose and treat.

### 1.3 Natural Remedies for Colorectal Cancer

According to World Health Organization (WHO), 75–80% of the world’s population solely rely on traditional medical systems for their first line of treatment due to concerns about the safety and efficacy of synthetic drugs (Hossain et al., 2014; Urbi and Zainuddin, 2015; Hossain and Urbi, 2016; Hossain M. S. et al., 2021). On the contrary, for being comparatively safe, natural products have gained tremendous importance as sources of polypharmacological drugs for infectious diseases, cancers, and neurological disorders (Hossain S. et al., 2021; Farooq et al., 2021). Moreover, indications of the importance of plants for diverse ailments in religious scripts attracted more researchers focusing on evaluating the scientific validity of traditional claims (Hossain et al., 2014; Hossain, 2016; Hossain et al., 2016). So, finding safer alternatives to systematic chemotherapeutic drugs from natural sources are an important and worthy study.

Herbs and spices have been commonly used as condiments to enrich aroma, taste, and colour for thousands of years. Even though they are consumed in small amounts, these herbs and spices contain many bioactive compounds and beneficial health effects. The role of spices and herbs in the inhibition of CRC cells growth has been reported in many recent studies (Zheng et al., 2016; Jaksevicius et al., 2017; Aasim et al., 2018; DeLuca et al., 2018; Khor et al., 2018; Wani and Kumar, 2018; Aiello et al., 2019; Rajasekaran, 2019; Buhrmann et al., 2020; Dandawate et al., 2020; Ganesan et al., 2020; Hallajzadeh et al., 2020; Hu et al., 2020; Malmir et al., 2020; Martínez-Aledo et al., 2020;; Pricci et al., 2020; Badsha et al., 2021; Kammath et al., 2021; Karthika et al., 2021). There is increasing evidence of preventing CRC by consuming fruits and vegetables, while red meat enhances the risk factors (Hallajzadeh et al., 2020). Similarly, dietary fibre was contradictory until recent findings showed that high dietary fibre could prevent cancers, including CRC (DeLuca et al., 2018; Masrul and Nindrea, 2019; O'Keefe, 2019). For example, consumption of one to three tablespoons of ground flaxseeds (FS) per day (8–24 g/day) has been suggested as part of a healthy eating pattern that works as a chemotherapeutic agent against CRC development (DeLuca et al., 2018).

**FIGURE 1:**
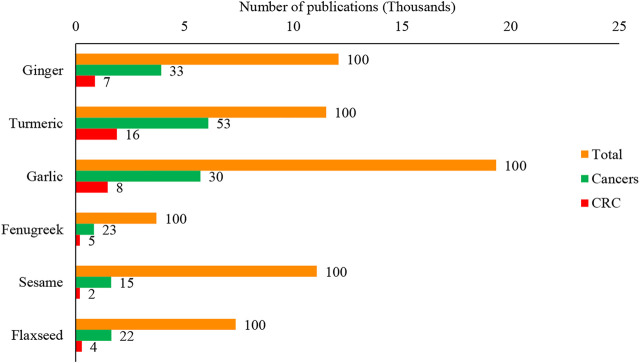
Scientific evaluation of culinary spices and herbs for the different research, including cancers and colorectal cancer. The value top on the bar represents the per cent (%) value of the total publications of each plant. The data were retrieved from the Scopus database on 15 August 2021 using the search keywords: Ginger OR *Zingiber officinale* Roscoe, Turmeric OR *Curcuma longa* L., Garlic OR *Allium sativum* L., Fenugreek OR *Trigonella foenum-graecum* L., Sesame OR *Sesamum indicum* L., Flaxseed OR *Linum usitatissimum* L., Cancer*, “Colorectal cancer” OR “Colon cancer”. CRC: Colorectal cancer.

Since many disease conditions, including CRC, commonly treated with culinary herbs and spices in traditional medical systems, are considered self-limiting, their purported benefits need critical evaluation intended for CRC management. This would be a worthy study for the community, particularly clinical practitioners and CRC patients. Therefore, in this study, six commonly used herbs and spices: ginger (*Zingiber officinale* Roscoe), turmeric (*Curcuma longa* L.), garlic (*Allium sativum* L.), fenugreek (*Trigonella foenum-graecum* L.), sesame (*Sesamum indicum* L.), and flaxseed (*Linum usitatissimum* L.) were chosen to evaluate their chemoprotective chemotherapeutic roles in CRC management critically and explored the possibility of developing these agents as anti-CRC pharmaceuticals. Scientific evaluation of these plants against cancers, particularly CRC, is increased over the last few years, and turmeric is the most extensively used spice evaluated for CRC (Figure 1).

Initially, this study comprehensively discussed the molecular basis of CRC development, followed by culinary and traditional uses, current scientific research, and publications of selected herbs and spices on cancers and their role in CRC management with underlying molecular mechanisms of action. Lead compounds have been discussed comprehensively for each herb and spice, including anti-CRC phytoconstituents, antioxidant activities, anti-inflammatory properties, and finally, anti-CRC effects with treatment mechanisms. Future possible works have been suggested where applicable.

## 2 Molecular Basis of Colorectal Cancer

Cancer is a genetic illness caused by oncogene activation, tumour suppressor gene dysfunction, or environmental mutagenesis (Imran et al., 2017). The effective control of cancer solely lies in a better understanding of its pathophysiology, and significant progress has been achieved in comprehending the molecular basis of cancer. Genetic and epigenetic changes play a role in the onset of neoplastic transformation of the healthy epithelium into malignant phases (Gorga, 1998). Progression of CRC is mainly involved with the silencing of tumour suppressor genes and activation of an oncogene (Malki et al., 2020).

As mentioned earlier, both genetic and epigenetic changes in the key genes are responsible for CRC development. This alteration is involved with three major pathways: CIN, MSI, and CIMP pathways (Pino and Chung, 2010). The CIN route is responsible for most CRC cases. This pathway is characterized by widespread abnormalities in chromosomal number (aneuploidy) and loss of heterozygosity. It can be caused by errors in chromosome segregation, telomere stability, or the DNA damage response, though the genes involved are currently unknown (Pino and Chung, 2010). Fearon and Vogelstein described the first multistep genetic model of colorectal tumorigenesis in 1990 (Fearon and Vogelstein, 1990). Later in 2010, Pino and Chung (Pino and Chung, 2010) critically evaluated the CIN pathway of CRC and discussed the role of each gene involved with CRC progression.

**FIGURE 2:**
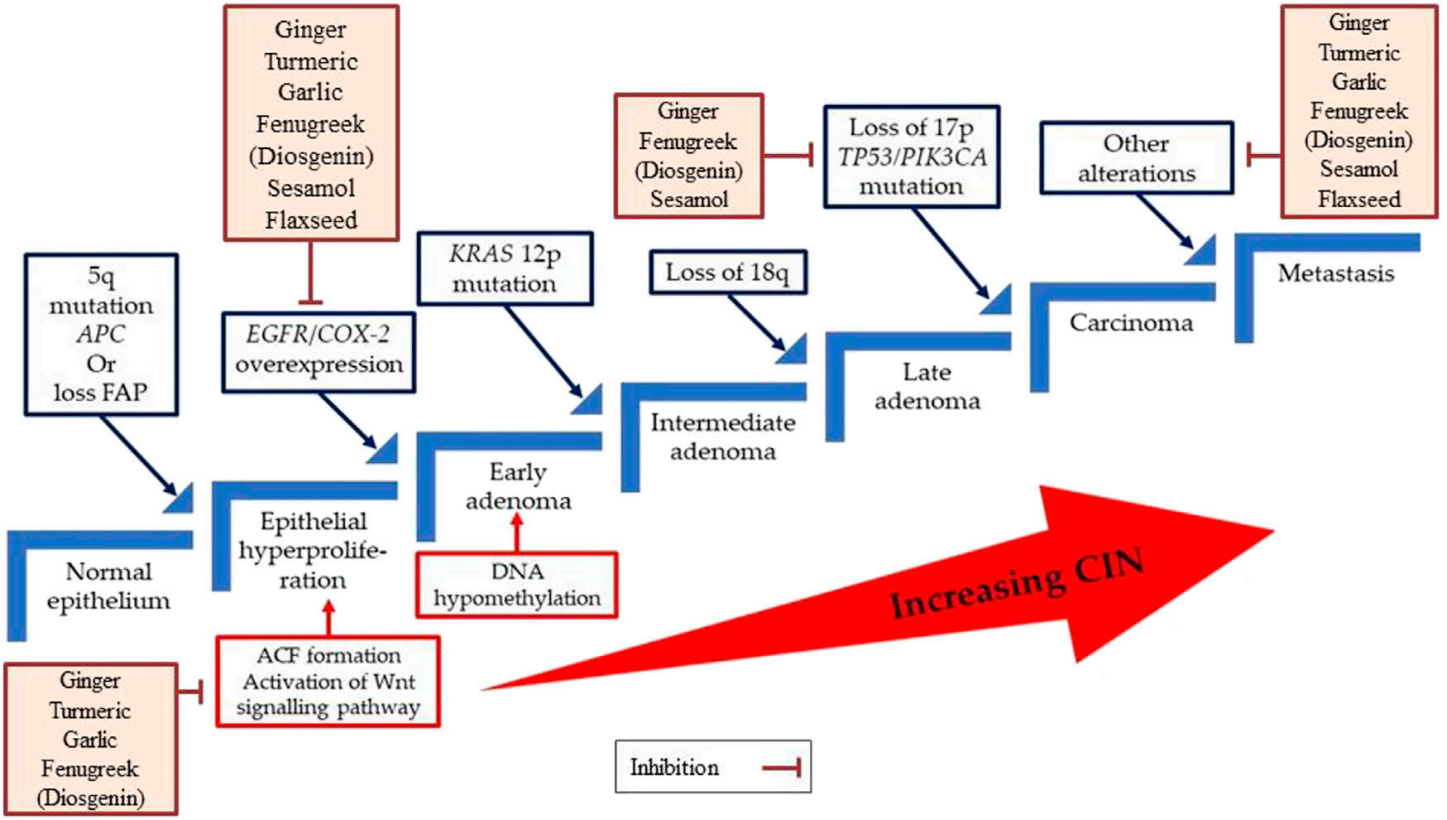
A multistep genetic model of colorectal carcinogenesis sequence. ACF, Aberrant crypt foci; APC, Adenomatous polyposis coli, CIN, Chromosomal instability, COX-2, Cyclooxygenase-2; EGFR, Epidermal growth factor receptor; FAP, Familial adenomatous polyposis.

According to the model proposed, the formation of aberrant crypt foci (ACF) is the initial step of CRC progression. Inactivating the *adenomatous polyposis coli* (*APC*) tumour suppression gene through the mutations can activate the Wnt signalling pathway at this stage. Subsequently, activating mutations in the proto-oncogene *KRAS*, mutations in the tumour suppressor gene *TP53*, as well as loss of heterozygosity at chromosome 18q are required for progression to larger adenomas and early carcinomas. In a tiny fraction of colorectal tumours, mutational activation of the *PIK3CA* gene occurs late in the adenoma-carcinoma sequence. Consistent with the evolution of adenomas that are not malignant, CIN is detected in benign adenomas and increases in tandem with tumour progression (Figure 2).

Nuclear Factor-kappa B (NF-κB) is a ubiquitous transcription factor regulating gene expression of inflammatory and immunological cytokines, cytokine receptors, and adhesion molecules in various cell signalling pathways. NF-κB activation also affects the control of apoptotic pathways, cell proliferation, differentiation, migration, angiogenesis, and tumour cell resistance to chemo/radiotherapy (Soleimani et al., 2020). NF-κB binds to an inhibitor, I-kappa B (IκB), present in the cytoplasm of most the quiescent cells and inactivates NF-κB by covering the nuclear localization sequence, blocking DNA binding and nuclear uptake of NF-κB (Baeuerle and Henkel, 1994). However, extracellular stimuli such as oncogenic molecules, and chemo/radiotherapy, cell surface receptors, including tumour necrosis factor receptors, interact with their specific ligands to cause an upregulation of the IκB kinase complex, which triggers down-stream genes expression that potentially promotes inflammation and cancer initiation/progression (Soleimani et al., 2020). In addition, active NF-κB in tumours with wild type *Kirsten Rat Sarcoma Virus* (*KRAS*) and *KRAS* mutations increased the activity of NF-κB signalling in patients with *KRAS* mutations, and patients exhibited a lower survival and weaker response to first-line treatment compared to other cases (Lin et al., 2012a; Lin et al., 2012b). Therefore, the NF-κB signalling pathway plays a vital role in accelerating cell proliferation, cell survival, and inhibition of apoptosis.

The well-established molecular basis of cancer helps determine confirmatory biomarkers that can improve clinical outcomes in patients with CRC and increase the survival of patients with metastatic cancer. Chemopreventive or chemotherapeutic agents target those biomarkers for the best outcomes to control CRC. In other words, identified biomarkers, such as *KRAS and TP53* genes, can be targeted to prevent or control CRC as inhibition of the *KRAS* gene or activation of the TP53 gene modulate the normal function of cells; thus, cancerous cells cannot sustain growth. Additionally, inhibition of the NF-κB signalling cascade limits cell proliferation; therefore, targeting this cascade may lead to preventive measures, and novel treatment approaches against CRC.

In our current review, we have annexed six culinary herbs that have strong vigour to inhibit the adenomas cell, carcinomas cell, and even several cancerous cell lines such as colorectal cancer, breast cancer, and prostate cancer (Matsuura et al., 2006). Ginger and its components may operate as chemopreventive agents by lowering COX-2 expression, according to *in vitro* and animal studies (Kim et al., 2005; Citronberg et al., 2013)(Kim et al., 2005; Citronberg et al., 2013). Gingerol works by activating key cell-signalling regulators and pathways such as Bax/Bcl2, p38/MAPK, Nrf2, p65/NF-B, TNF-, ERK1/2, SAPK/JNK, ROS/NF-B/COX-2, caspases-3, -9, and p53 (Wee et al., 2015; de Lima et al., 2018). Turmeric extract suppresses metastasis by regulating several targets, including molecules involved in the Wnt and Src pathways, EMT, and EGFR-related pathways. It also restricts FAK/Src, STAT3, Erk, and Akt pathways suppressing cell proliferation, motility, and migration (Li et al., 2018; Li et al., 2021). Garlic and its constituents suppress tumour biomarker aberrant crypt foci (ACF), NF-κB, anti-apoptotic genes (Bcl-2, cIAP1/2, and XIAP), and inflammatory genes (iNOS and COX-2), and EGFR, whereas it induces apoptotic gene expression (Ban et al., 2007; Ngo et al., 2007; Saud et al., 2016; Mondal et al., 2022). A few studies found a significant reduction of ACF with 1% fenugreek or 0.1% or 0.05% diosgenin, which is also implicated in the suppression of COX-2 as well as the stimulation of nuclear factor-B, p53, and p21 expression (Moalic et al., 2001; Raju et al., 2004). Sesamol (100 µM) inhibits the expression of COX-2 and cytosolic prostaglandin E2 synthase mRNA in polyp sections and targets the p53, MAPK, JNK, PI3K/AKT, TNFα, NF-κB, PPARγ, caspase-3, Nrf2, eNOS, and LOX signalling pathways (Shimizu et al., 2014; Majdalawieh and Mansour, 2019). Flaxseed meal elevates the mitochondrial apoptosis genes such as p53 and cyclin-dependent kinase inhibitor 1A (p21) as well as cell cycle arrest genes (Hernández-Salazar et al., 2013). Furthermore, the COX-1 and COX-2 protein level in the colonic tissue is considerably reduced (Bommareddy et al., 2006).

### 3 Correlation of Oxidative Stress, Inflammation, and Carcinogenesis

**FIGURE 3:**
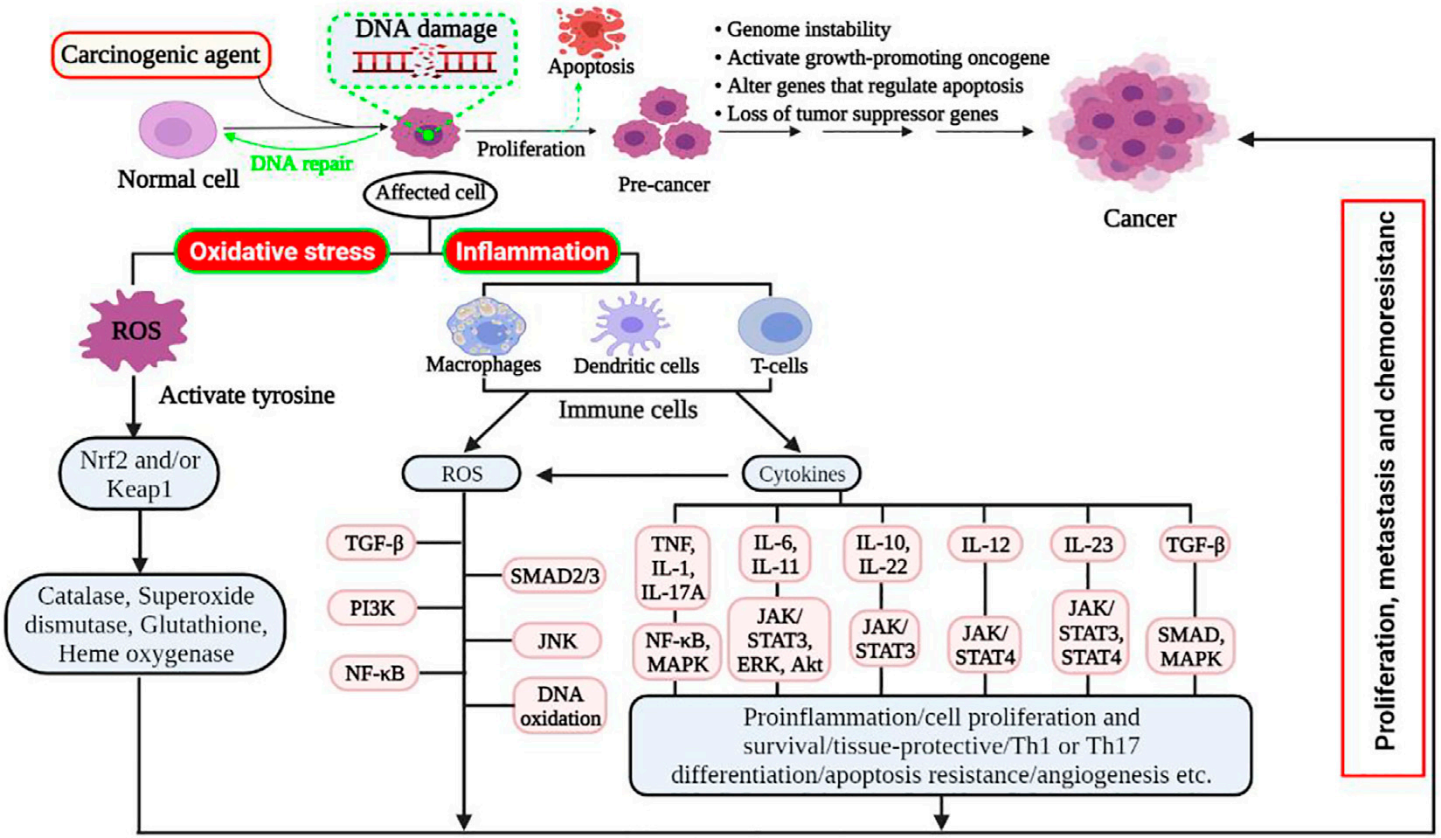
Schematic mechanism of oxidative stress and inflammation-induced cancer development. Damaged cells or tissues produce ROS resulting in oxidative stress and/or inflammation. Oxidative stress: regenerated ROS can be detoxified with the presence of balanced detoxifying agents, such as antioxidants. However, excessive ROS induces apoptotic signalling pathways and promotes carcinogenesis in cells with faulty signalling by deregulating biomolecules. Hence, it targets *Nrf2* and its regulator *Keap1* and downregulates antioxidant enzymes that result in high intracellular ROS levels, which induce cell proliferation, metastasis, and chemoresistance by rescuing *Nrf2* transcription. Inflammation: various diseases and stress conditions causes inflammatory cell infiltration that induces ROS and different cytokines. The elevated ROS activates latent TGF-complex, which binds to its receptor and activates signalling pathways such SMAD2/3, PI3K, and JNK. It also activated tyrosine kinase that allowed NF-κB (active form) to enter the nucleus, further activating target genes for chemokines, cytokines, adhesion molecules, and receptors to cause cell proliferation, growth, and differentiation. Akt, protein kinase B; Erk, extracellular signal-regulated kinase; IL, interleukin; JNK, c-Jun N-terminal kinase; Keap1, kelch-like ECH (enoyl-CoA hydratase)-associated protein 1; MAPK, mitogen-activated protein kinase; Nrf2, nuclear-related factor 2; NF-κB, nuclear factor-kappa B; Pi3K, Pi3 kinase; ROS, reactive oxygen species; STAT3, signal transducer and activator of transcription 3; SUZ12, suppressor of zeste 12; TGF, transforming growth factor; TNF, tumour necrosis factor.

Reactive oxygen species (ROS) are produced after exposure to different physical agents, including ultraviolet rays and heat, as well as after chemotherapy and radiotherapy in cancer treatment. Over the past few decades, researchers have realized that ROS plays a significant role in the aetiology of various diseases, including cancer, cardiovascular disease, and inflammation injury. Excessive production of ROS in cellular life needs to be regulated tightly. Because ROS have the potential to initiate the degenerative process in cells; however, living organisms have several antioxidant systems that scavenge the adverse effects of ROS on cells (Karker et al., 2016). These drastic impacts on cells include oxidative stress, damaging biomolecules, including DNA, lipid oxidation, protein degradation, and cellular apoptosis (Figure 3) (Valko et al., 2007).

ROS are by-products of regular cellular metabolism that play crucial roles in activating signalling pathways in cellular life (Perillo et al., 2020). When cells or tissues are exposed to prolonged environmental stress, ROS are created over an extended period, resulting in irreversible damage to cell structure and function, as well as the induction of somatic mutations and neoplastic transformation (Khandrika et al., 2009). Indeed, oxidative stress is associated with cancer onset and development, either by increasing DNA mutations or generating DNA damage, genome instability, and cell proliferation (Figure 3) (Fang et al., 2009; Visconti and Grieco, 2009). Additionally, proteins and lipids are also key oxidative targets, and altering these molecules increases the risk of mutagenesis (Schraufstätter et al., 1988). The adverse effects of ROS can be tightly controlled through a sophisticated enzymatic antioxidant system [e.g., superoxide dismutase (SOD), glutathione peroxidase (GPx), glutathione reductase, and catalase] (Soraya and Mozafar, 2018).

Chronic inflammation is caused by various biological, pharmacological, and physical factors, and it has been linked to an elevated risk of numerous types of cancer in humans, including CRC. Epidemiological and experimental evidence suggests that oncological illnesses like cancer have been linked to this inflammation (Reuter et al., 2010). This inflammation is now regarded as a “secret killer” for diseases such as cancer. For example, inflammatory bowel diseases such as Crohn’s disease and ulcerative colitis are associated with an increased risk of colon adenocarcinoma, and chronic pancreatitis is related to an increased rate of pancreatic cancer (Reuter et al., 2010).

An elevated ROS is produced in the inflammatory cells due to increased oxygen absorption in the damaged area. As a result of the increased ROS, the latent TGF-complex is activated, which binds to its receptor and activates different signalling pathways, such as SMAD2/3, PI3K, MAPK/AP-1, and JNK. (Coussens and Werb, 2002). In addition, inflammatory cells also generate soluble mediators, such as cytokines and chemokines, which persuade changes in transcription factors and can trigger different signal transduction cascades, including NF-κB, STAT3, and activator protein-1 (AP-1), Nrf2 (Figure 3). The aberrant expression of inflammatory cytokines like TNF, different interleukins (*i.e.*, IL-1, IL-6, IL-10, IL-11, IL-12, IL-22, IL-23), and chemokine IL-8 have also been reported to play a pivotal role in oxidative stress-induced inflammation (Kanda et al., 2017). Therefore, this sustained inflammatory/oxidative stress leads to damage to neighbouring healthy epithelial and stromal cells, and prolonged time may lead to carcinogenesis because of genome instability, activate of the growth-promoting oncogene, alteration of genes that regulate apoptosis, and loss of tumour suppressor genes (Federico et al., 2007; Liu et al., 2021).

## 4 Therapeutic Potential of Culinary Herb and Spice for Colorectal Cancer Management

Food is consumed either raw or cooked to provide energy and nutritional support for an organism. In addition to enhancing taste, aroma, and colour, herbs and spices also provide nutritional value. Apart from their culinary uses, ginger, turmeric, garlic, fenugreek, sesame, and flaxseeds are traditionally used for different ailments, including cancers (Table 1).

**TABLE 1:** Traditional uses of herb and spice along with their scientific name, family, culinary uses, part used, and lead compounds.

Common name	Scientific name	Family	Type	Culinary use	Part used	Lead compound(s)	Traditional uses	References
Ginger	*Zingiber officinale* Roscoe	Zingiberaceae	Spice	Use for pungent flavour and taste in foods and beverages	Rhizomes, leaves	Gingerols, paradols, shogaols, quercetin	Common cold, digestive disorders, rheumatism, neuralgia, colic and motion sickness, migraines, hypertension, abdominal distension, dropsy, cancer, and diabetes	Mascolo et al. (1989), Surh et al. (1998)
Turmeric	*Curcuma longa* L.	Zingiberaceae	Spice	Used for a specific flavour and yellow colour	Rhizomes	Curcumin, calebin A	Rheumatoid arthritis, chronic anterior uveitis, conjunctivitis, skin cancer, smallpox, chickenpox, wound healing, urinary tract infections, liver ailments, digestive disorders; to reduce flatus, jaundice, menstrual difficulties, colic, abdominal pain and distension, and dyspeptic conditions	Dixit et al. (1988), Bundy et al. (2004), Prasad and Aggarwal, (2011)
Garlic	*Allium sativum* L.	Amaryllidaceae	Spice	Used for pungent flavour as a seasoning or condiment.	Bulb, leaves, flower	Diallyl sulfide, diallyl disulfide, diallyl trisulfide, diallyl tetra-sulfide, S-allyl mercaptocysteine, allicin, selenomethionine and se-methyl-L-selenocysteine	Typhus, dysentery, cholera, influenza maintain and increase their strength, abnormal growths, circulatory ailments, general malaise and infestations with insects and parasites, alleviation of joint disease and seizures	Rivlin, (2001), Ayaz and Alpsoy, (2007), Petrovska and Cekovska, (2010)
Fenugreek	*Trigonella foenum-graecum* L.	Fabaceae	Spice	Used as leafy vegetables and seasonings	Seed, leaves	Diosgenin	Menstrual pains, sedating tummy, boost physique, to treat weakness of body, gout, breast milk stimulant, tonic, digestive and respiratory problems, and ease childbirth	Yoshikawa et al. (1997), Bahmani et al. (2016), Wani and Kumar, (2018)
Sesame	*Sesamum indicum* L.	Pedaliaceae	Herb		Seed	Sesamol	Benefits the liver, kidney, spleen, and stomach, lubricates the intestines, nourishes all the internal viscera, blackens the hair, kills intestinal worms such as Ascaris, tapeworm	Anilakumar et al. (2010), Pathak et al. (2014)
Flaxseeds	*Linum usitatissimum* L.	Linaceae	Herb	Used as a featured ingredient in cereals, pasta, whole-grain bread and crackers, energy bars, meatless meal products, and snack foods	Seed, leaves	Linolenic acid, lignans, p-coumaric and ferulic acid	Dyspnoea, asthma, dysphonia, bad cough, bronchitis, constipation, pulmonary tuberculosis, hemoptysis, splenomegaly, and stomach ulcer	Goyal et al. (2014)

**FIGURE 4:**
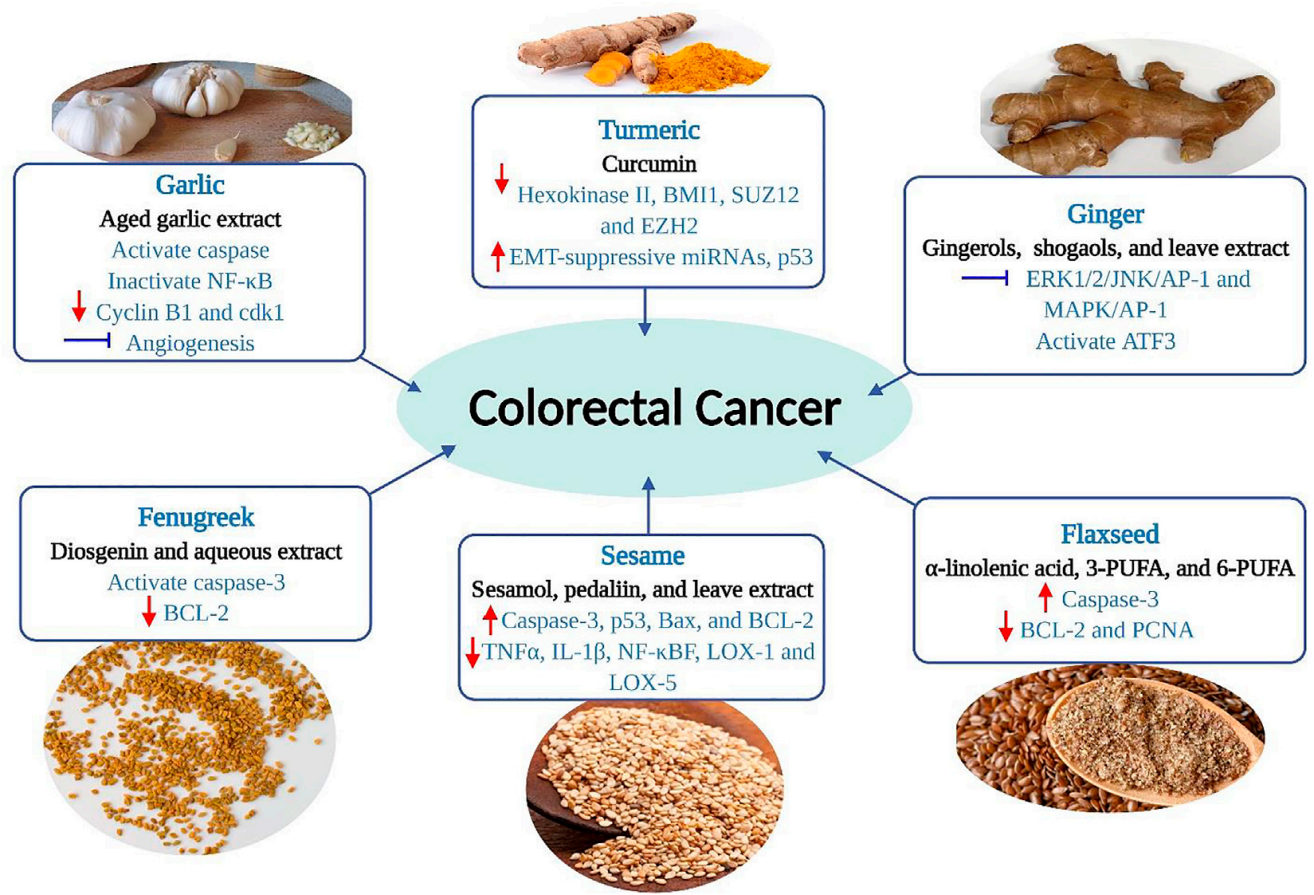
Chemopreventive effects of selected herbs and spices against colorectal cancer by targeting diverse mechanisms of action. The red arrow (up) denotes stimulation or up-regulation, the red arrow (down) denotes down-regulation, and a bar perpendicular to the end of the line (blue) denotes inhibition. AP-1, activator protein 1; ATF3, activating transcription factor 3; Bax, Bcl-2 associated X protein; BCL-2, B-cell lymphoma-2; cdk1, cyclin-dependent kinase 1; BMI1, B cell-specific Moloney murine leukemia virus integration site 1; COX-2, cyclooxygenase 2; EMT, epithelial-to-mesenchymal transition; Erk, extracellular signal-regulated kinase; EZH2, enhancer of zeste homolog 2; JNK, c-Jun N-terminal kinase; LOX, lipoxygenase; MAPK, mitogen-activated protein kinase; NF-κB, nuclear factor-kappa B; p53, tumor protein p53; PCNA, proliferating cell nuclear antigen; PUFA, polyunsaturated fatty acids; SUZ12, suppressor of zeste 12; TNF, tumor necrosis factor.

These plants are widely investigated for the scientific validity of health benefits or traditional uses, particularly their anti-cancerous role. The extracts or compounds that possess antioxidants showed potential anti-cancerous effects. Antioxidants are substances that, when present in low concentrations compared to the substrate, prevent or delay the oxidation of the substrate. On the other hand, the substrate would otherwise be oxidized by the pro-oxidants. Different parts, plant extracts, and isolated compounds of the selected herbs and spices have potential antioxidant properties that show anti-inflammatory and anti-cancer effects. An overview of their chemopreventive or chemotherapeutic role in CRC management by targeting diverse mechanisms of action is shown in Figure 4.

### 4.1 Ginger

The botanical name of ginger is *Zingiber officinale* Roscoe, which belongs to the family Zingiberaceae. It is a herbaceous perennial flowering plant that originated in Southeast Asia. It is one of the most consumed dietary condiments globally and is now produced worldwide, including in Bangladesh, India, China, Nigeria, Nepal, Indonesia, and Japan (Surh, 1999). The rhizome, the horizontal stem from which the roots grow, is the central portion of ginger that is widely used and consumed in numerous forms, such as fresh, dried, pickled, preserved, crystallized, candied, powdered or ground (Bode and Dong, 2011).

**FIGURE 5:**
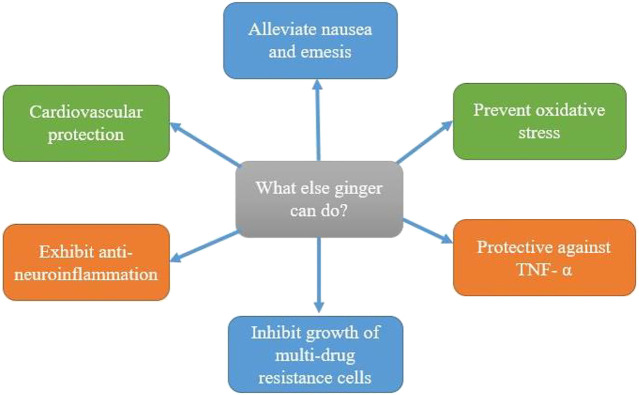
Health benefits and anticancer properties of ginger.

Ginger is an excellent source of antioxidants used to treat ailments from colds to cancer (Bode and Dong, 2011). The popularity of ginger for scientific research has surged in recent years. As of 15 August 2021 (Scopus database), approximately 12,092 papers with a focus on the beneficial effects of ginger have been published between 1853 and 2021. The papers focused on only cancer is 33%, while 7% focused mainly on CRC (Figure 1). This plant has many other health benefits related to cancers (Figure 5).

#### 4.1.1 Lead Compounds of Ginger

**FIGURE 6:**
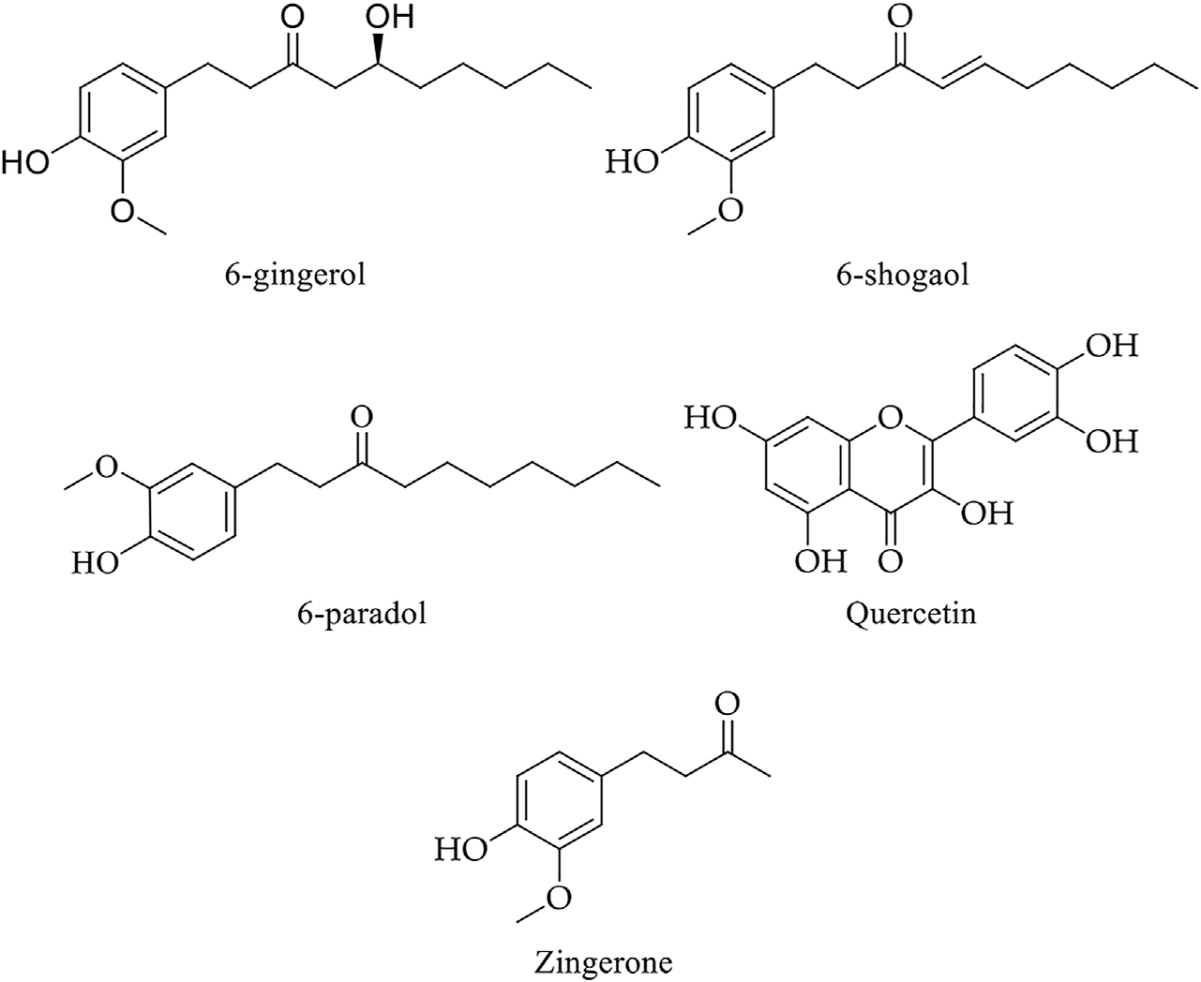
Chemical structure of main non-volatile bioactive compounds of ginger.

Ginger has at least 115 compounds (volatile and non-volatile) in fresh and dried rhizomes identified in different extracts. Among these, gingerols, parasols, shogaols, and quercetin are the most common constituents and exert various powerful therapeutic and preventive effects (Bode and Dong, 2011). Examples of volatile components are hydrocarbons, zingiberene, sequiphellandrene, α-curcumin, and other sesquiterpenes, while non-volatile pungent phenolic compounds are used 6-gingerol, 6-shogaol, 6-paradol, quercetin, and quercetin zingerone (Figure 6) (Vedashree et al., 2020). The non-volatile compounds exert chemopreventive and therapeutic efficacy (Figure 4 and Table 2) (Fu et al., 2014; Park et al., 2014; Radhakrishnan et al., 2014).

**TABLE 2:** An overview of cytotoxic effects of culinary herbs and spices on colorectal cancer.

Name	Extract/ compound	Cell line	Cellular effect	Mechanism	References
Ginger	Gingerols, shogaols, and leave extract	HCT116, SW480, LoVo	Inhibit cell proliferation and induce apoptosis in CRC, but not in normal colorectal cells Inhibit the growth of cells and induce apoptosis Prevent PMA-induced proliferation in CRC	Inhibit ERK1/2/JNK/AP-1 pathway Activate ATF3 promoter and increase ATF3 expression Inhibit MAPK/AP-1 signalling	Fu et al. (2014), Park et al. (2014), Radhakrishnan et al. (2014)
Turmeric	Curcumin extract	HT29 HCT 116 Colon 26-M01	Inhibit production of mucosal concentrations of pro-carcinogenic eicosanoids 5-HETE and PGE-2 PGE-2 could reverse induced apoptosis Inhibit the growth of hCAC Curcumin+5-FU enhance cellular apoptosis and inhibit proliferation in 5-FU resistant cells Decrease cell motility and migration	G0/G1 phase arrest, down-regulation of cell cycle progression Down-regulate expression of hexokinase II Induced dissociation of hexokinase II from the mitochondria led to mitochondrial-mediated apoptosis Upregulate EMT-suppressive miRNAs in 5-FU resistant cells Down-regulate BMI1, SUZ12, and EZH2 transcripts Upregulating p53 molecule expression Multiple signalling pathways such as AKT, Erk, and STAT3 inhibit colony formation in murine colorectal cancer cells	Carroll et al. (2011), Manikandan et al. (2012), Shehzad et al. (2014), Guo et al. (2015), Toden et al. (2015), Wang et al. (2015), Rajitha et al. (2016), Li et al. (2018)
Garlic	AGE Aged garlic extract	DLD-1, Colo 205, HT29, SW480, SW620	Decrease ACF Showed a lower number of adenoma and adenocarcinoma lesions Suppressed the proliferative activity in adenoma and adenocarcinoma lesions but showed no effect on normal colon mucosa Regulate ER-stress induce apoptosis (80% apoptosis) Inhibits angiogenesis and proliferation	Caspase activation Inactivation of NF-κB Delayed cell cycle progression by downregulating cyclin B1 and cdk1 expression *via* inactivation of NF-κB Prevent tumour formation by inhibiting angiogenesis through the suppression of endothelial cell motility, proliferation, and tube formation Increase cellular adhesion to collagen and fibronectin, and inhibit angiogenesis in the colorectal cancer cell.	Matsuura et al. (2006), Jikihara et al. (2015), Tung et al., 2015)
Fenugreek	Diosgenin and aqueous extract	HT29	Induce apoptosis Inhibit the production of AOM and induce ACF Reduce LPO and increase GPx, GST, SOD	Suppress BCL-2 and activate caspase-3 protein expression	Raju et al. (2004), Sushma and Devasena, (2010)
Sesame	Sesamol, pedaliin, and leave extract	HT29, HCT116	Induce apoptosis Induce G0/G1 and S-phase cell cycle arrest	Suppress TNFα and IL-1β expression, NF-κB signalling, and LOX-1 and 5-LOX activity Modulate caspase-3, p53, Bax, and BCL-2 expression	Gupta et al. (2009), Chu et al. (2010a), Chu et al. (2010b), Wu et al. (2015), Kim et al. (2021)
Flaxseed	α-linolenic acid, 3-PUFA, and 6-PUFA Extract (Oil)	CaCo-2, SW480, Colo 201 LoVo RKO	Inhibit cell proliferation and induce apoptosis Induce S-phase cell cycle arrest, elevate cyclin A protein levels, and increase the proportion of apoptotic cells Mitochondrial disfunction and trend to apoptosis	Upregulate Caspase-3 Down-regulate BCL-2 and PCNA protein Augmenting ROS production, accumulating intracellular ca^2+^ decreasing mitochondrial membrane potential and production of ATP	Danbara et al. (2005), Bommareddy et al. (2010), Chamberland and Moon, (2015), Zhang et al. (2015)

5-HETE, 5-hydroxyeicosatetraenoic acid; AGE, Aged garlic extract, cdk1, Cyclin-dependent kinase 1; PGE-2, Prostaglandin E-2; hCAC, Human colon adenocarcinoma cell lines; EMT, Epithelial-mesenchymal transition; AOM, azoxymethane; ACF, aberrant crypt foci; LPO, Plasma lipid peroxides; GPx, Glutathione peroxidase, GST, Glutathione S-transferase; SOD, Superoxide dismutase; NF-κB, Nuclear Factor-kappa B; PCNA, proliferation cell nuclear antigen; PUFA, Polyunsaturated fatty acid.

#### 4.1.2 Antioxidant Activity of Ginger

Ginger extracts, powder, and constituents have shown potential antioxidant activity *in vitro* and *in vivo* models (Kikuzaki and Nakatani, 1993; Stoilova et al., 2007; Wang et al., 2018; Tanweer et al., 2020; Naliato et al., 2021). These studies showed that solvent has significant effects on the effectiveness of antioxidant properties. Aqueous ethanolic solution (0.02%) showed high antioxidant activity (Kikuzaki and Nakatani, 1993). The solution was prepared from different extracts of ginger like dichloromethane, methanol, and α-tocopherol. The dichloromethane extract exhibited higher activity than α-tocopherol and methanol extract. The ginger extract inhibited the hydroxyl radicals 79.6% at 37°C and 74.8% at 80°C, which showed higher antioxidant activity than quercetin and chelated Fe^3+^ in the solution (Stoilova et al., 2007). Gingerol related compounds substituted with an alkyl group bearing 10-, 12- or 14-carbon chain length might contribute to both radical scavenging effect and inhibitory effect of autoxidation of oils; however, there was no significant difference in the activity among the compounds with different alkyl chain length (Masuda et al., 2004). These results suggested that the antioxidant action may be attributed to radical scavenging and substrate affinity. Ginger bioactive compounds can also stimulate a plethora of enzymes, such as glutathione reductase, glutathione S–transferase, and glutathione peroxidase, that help mitigate free radicals that induce oxidative stress startlingly suppress colon carcinogenesis (Manju and Nalini, 2005). Consumption of ginger extracts may reduce or delay the progression of diseases that oxidative stress occurs due to a lack of antioxidant supplementation (Tohma et al., 2017) because ginger cakes or bread showed high antioxidants activity by scavenging peroxyl radicals (Martinez-Villaluenga et al., 2009; Balestra et al., 2011; Ademosun et al., 2021). Therefore, it can assume that ginger-based bakeries or beverages would be effective functional dietary products in managing and preventing cancers.

#### 4.1.3 Anti-Inflammatory Effects of Ginger

Phytocompounds isolated from ginger, such as gingerol and shogaol, can suppress the synthesis of pro-inflammatory cytokines such as IL-1, IL-8, and TNF- α. (Tjendraputra et al., 2001). An NF-κB signalling pathway is linked with chronic inflammatory diseases like cancer, allergy, myocardial infarction, asthma, arthritis, multiple sclerosis, and atherosclerosis. Habib et al. (2008) demonstrated that the ginger extract has the magnificent potential to lessen the expression of the NF-κB signalling pathway. Cyclooxygenases (*i.e.*, COX-1, COX-2) enhance prostaglandin-mediated inflammation. Gingerol impedes COX-2 expression induced by lipopolysaccharides (LPS) (Lantz et al., 2007). A double-blind, placebo-controlled, randomized experiment reported that daily consumption of raw and heat-treated ginger (2 g) for 11 consecutive days resulted in moderate-to-large reductions in muscle pain compared to the placebo (Black et al., 2010). In LPS induced inflammation, one of the lead compounds of ginger, [6]-shogaol reduced the levels of nitric oxide synthases (iNOS), COX-2, and phospho-NF-kB, suppressed histone deacetylase-1 (HDAC-1) expression, and increased histone H3 acetylation expression. [6]-Shogaol can inhibit HDAC-1 expression, comparable to that of commonly used HDAC inhibitors Trichostatin A and MS275 (Shim et al., 2011). This result indicates that a ginger supplement rich with [6]-shogaol could significantly attenuate various inflammatory responses.

#### 4.1.4 Anti-Colorectal Cancer Effects and Mechanisms of Actions of Ginger

Ginger leaves extract induced apoptosis in human colorectal cancer cells, HCT116, SW480 (human colon adenocarcinoma cells), and LoVo by activating transcription factor 3 (ATF3). ATF3 is responsible for the induction of apoptosis in CRC cells by regulating the ERK1/2 pathway, where ginger leaves (50, 100, and 200 μg/ml for 24 and 48 h) interact with the cAMP-responsive element-binding (CREB) site and activate ATF3 (Tang et al., 2002; Hsu et al., 2005; Park et al., 2014). Another investigation reported that ginger extract inhibited CRC cell growth (HCT-116) by down-regulating the K-ras and MMP-2 marker gene expressions. K-ras is crucial in colorectal metastasis by regulating VGEF, protease expression, apoptosis, adhesion, and motility (Lavrado et al., 2015).

### 4.2 Turmeric

Turmeric (*Curcuma longa* L.) is also derived from the Zingiberaceae family. In addition to improving taste, turmeric was one of the spices used to preserve food. Yellow turmeric rhizomes give an aromatic flavor and slightly bitter taste. (Dhakal et al., 2019). Turmeric rhizomes are an excellent antioxidant source, and it has free-radical scavenging properties (Restrepo-Osorio et al., 2020). Its extracts (1–2%) are considered a natural preservative and free from microbial contamination at least for 90 days of storage (Gul and Bakht, 2015). Turmeric is generally given at a dose of 5–500 mg/kg for nutritional purposes depending on the food categories like dairy products, beverages, cereals, mustard, food concentrates, pickles, sausages, confectionery, and ice cream, meat, fish, eggs, and other confectionaries. It is also mixed with other compounds, including annatto, seasonal sauces, mayonnaise, and butter (Sharifi-Rad et al., 2020). Turmeric is rich in polyphenols and universally known as the “wonder drug of life.” The lead compound, curcumin, is responsible for a wide range of pharmacological activities, including antioxidant, anti-cancer, anti-arthritic, anti-microbial, anti-diabetic, anti-inflammatory activities, and avails in the treatment of many ailments, including tendinitis, liver cirrhosis, Alzheimer’s disease, heart attack, hypoglycemia, gastrointestinal problems, worms, swelling, cancer, skin and ocular perceiver infections (Gera et al., 2017). Currently, the major focus has been given by scientists on cancer. As of 15 August 2021 (Scopus database), the effect of turmeric/curcumin on cancer; between 1881 and 2021, was the topic of 11,519 papers, with 53% related to cancer and 16% associated exclusively on CRC (Figure 1). The wide application of curcumin in diverse fields had caused its global market to expand exponentially. The pharmaceutical industry, particularly those focused on anti-cancer medication formulations, is the most significant application category, accounting for more than half of the global market, followed by the food and cosmetic industries (Sharifi-Rad et al., 2020).

#### 4.2.1 Lead Compounds of Turmeric

**FIGURE 7:**
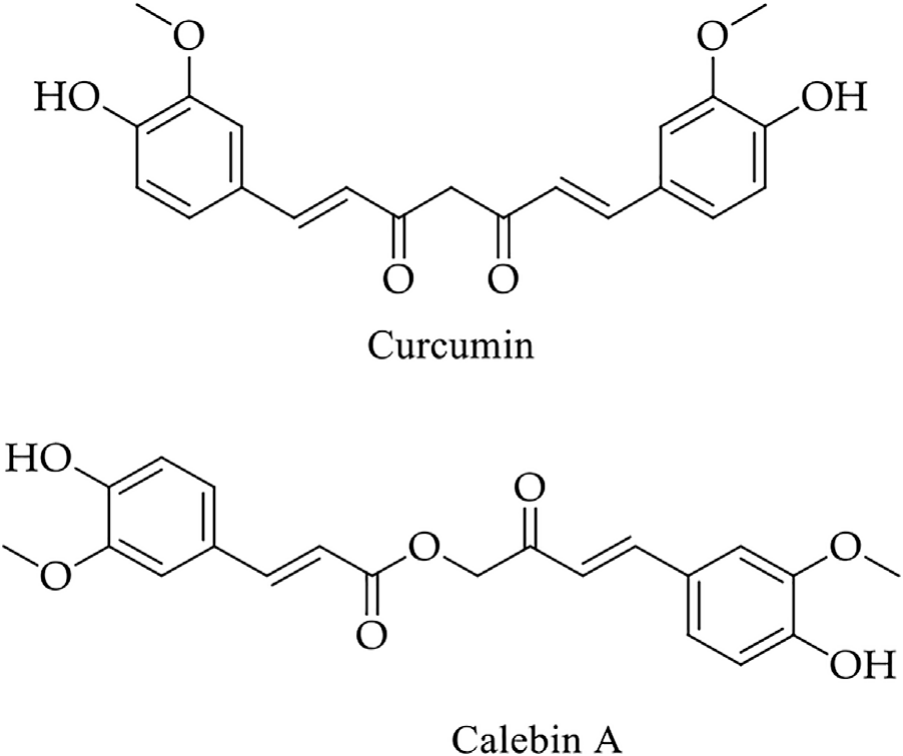
Chemical structure of main bioactive compounds of turmeric.

Turmeric powder contains various bioactive components. Dry turmeric contains 69.43% carbohydrates, 6.3% proteins, 5.1% oils, 3.5% minerals, and other elements (15.67%). Curcumin and calebin A are lead compounds (Figure 7) with a magnificent biological role in CRC patient management (Figure 4 and Table 2).

#### 4.2.2 Antioxidant Activity of Turmeric

Turmeric can mitigate the rise of free radical formation in the living cells responsible for damaging the biomolecules, such as lipid, protein, and DNA (Goud et al., 1993). A study reported by Tilak et al. (2004) showed that turmeric, as used in cooking and in-home remedies, and its major compounds have significant antioxidant abilities at different levels of action. This study prepared six different types of standardized aqueous and ethanol extracts using the processed powder or raw turmeric rhizome as per the cooking mood. The ethanol contains more phenolic and flavonoid content than aqueous extracts. In all antioxidant tests using raw and processed turmeric, ethanol extracts were performed over the aqueous extracts. Boiled ethanol extract (10 min) had the strongest activity in two chemical assays - ferric reducing antioxidant power (FRAP) and 1,1-diphenyl-2-picryl hydroxyl (DPPH) radical scavenging test. However, in the ferryl myoglobin assay, ethanol extracts (raw turmeric stirring in ethanol for 1 h) exhibited the highest total antioxidant activity (TAA). Boiling the aqueous extracts increased their potency compared to the aqueous extracts. Turmeric boiling extracts were more efficient at scavenging 2,2-azobis-3-ethylbenzthiazoline-6-sulfonic acid (ABTS) radicals than aqueous turmeric extracts. There is no doubt numerous studies reported on the antioxidant activity of turmeric extracts and their different isolated compounds. At the same time, the *in vitro* antioxidant activities of turmeric extracts have been supported by the *in vivo* studies to support its pharmacological applications (Sreekanth et al., 2003; Dall'Acqua et al., 2016; Mohammed et al., 2020). A proprietory formulation containing extract of turmeric obtained by supercritical carbon dioxide gas extraction and post-supercritical hydroethanolic extraction is known as Smoke Shield. Administration of Smoke Shield to mice increased antioxidant enzymes in blood, liver, and kidney (Sreekanth et al., 2003). Smoke Shield increased glutathione-S-transferase activities in the liver and kidney. Additionally, it increases superoxide dismutase and glutathione, whereas it decreases glutathione peroxidase in smokers’ blood. Smoke Shield contains significant antioxidant action, inhibits phase I enzymes, and increases detoxification enzymes, making it a chemoprotective herbal preparation (Sreekanth et al., 2003). The oral administration of turmeric extract to healthy rats decreased urinary levels of allantoin, m-tyrosine, 8-hydroxy-2′-deoxyguanosine, and nitrotyrosine. This finding supports the *in vivo* antioxidant effect of turmeric (Dall'Acqua et al., 2016). Another recent *in vivo* antioxidant study of methanol extract of turmeric showed a significant decrease in SOD, catalase (CAT) and GPx levels in both liver and kidney of Alloxan-induced diabetic rats (Mohammed et al., 2020). These findings suggested that turmeric supplements could potentially neutralize the ROS level in cells even if used in cooking, either stirring on oil for 10 min or 30 min in aqueous; however, further investigation on the complex role of antioxidant curcumin effects is required before making a precise conclusion.

The main bioactive compound of turmeric, curcumin (42 µM) integrated with rat liver mitochondria, reduces ascorbate-Fe^2+^ driven lipid peroxidation significantly. During incubation (0–60 min), the per cent inhibition was almost 100% without any lag period of inhibition (Tilak et al., 2004). Another phytoconstituent of turmeric called turmeric was an effective antioxidant/DNA protectant/antimutagen. It has three methionine residues that are responsible for its antioxidant properties. Turmeric at a 183 nM is highly protective (80%) to membranes and DNA against oxidative injury. Additionally, it is noncytotoxic up to milligrams doses in human lymphocytes (Srinivas et al., 1992). Curcumin and turmeric controlled oxidative stress by reducing the level of thiobarbituric acid-reactive substances (TBARS) and protein carbonyls and repealing altered antioxidant enzyme activities in rat models (Suryanarayana et al., 2007). Many other studies also reported that turmeric extracts and their constituents have potential free radical scavenging activity, and ethanol extracts are more efficient than aqueous extracts (Cousins et al., 2007; Singh et al., 2010; Lim et al., 2011; Tanvir et al., 2017; Arumai Selvan et al., 2018). The formulation of turmeric extracts using modern technology like spray-dried microparticles (Martins et al., 2013), silver nanoparticles (Arumai Selvan et al., 2018), and puffing (Choi et al., 2019) enhance the antioxidant activity of turmeric.

#### 4.2.3 Anti-Inflammatory Effect of Turmeric

Traditionally, as an anti-inflammatory antidote in ayurvedic medicine, Turmeric powder is implicated as an anti-inflammatory antidote. In a rat model, the production of these enzymes was elevated by curcumin treatment (Tvrdá et al., 2016). In *the in-vitro* experiment, the macrophage-mediated inflammation due to ROS production has reduced by giving 10 μM curcumin (Amano et al., 2015). The increasing detrimental bacterial population in the colon so prompts to produce of carcinogenic chemicals, toxins that are responsible for the development of colon cancer; for instance, *Bacteroides fragilis* produces *Bacteroides fragilis* toxins (BFTs) that subsequently activate the STAT3 signalling pathway and stimulate IL-17 cytokines production that subsequently promotes NF- κB and Wnt signalling pathway activation leading to abnormal cell division (Chung et al., 2018). Caleb in A is a potent anti-inflammatory component of turmeric responsible for inhibiting cancer formation through this NF-κB signalling pathway (Buhrmann et al., 2020). Another investigation has demonstrated that the inflammation mediated by NF-κB activation is suppressed by averting IκBα kinase and AKT signalling pathway because of using curcumin (Aggarwal et al., 2006). The administration of curcumin is also thought to have reduced the expression of inflammatory cytokines, such as C-reactive protein, cyclooxygenase-2 (COX-2), TNF-, CXCR-4, MIP-1, IL-1, IL-6, and IL-8 (Gonzales and Orlando, 2008; Wang and Dubois, 2010; Kim et al., 2012; Afzali et al., 2021).

#### 4.2.4 Anti-Colorectal Cancer Effects and Mechanisms of Actions of Turmeric

**FIGURE 8:**
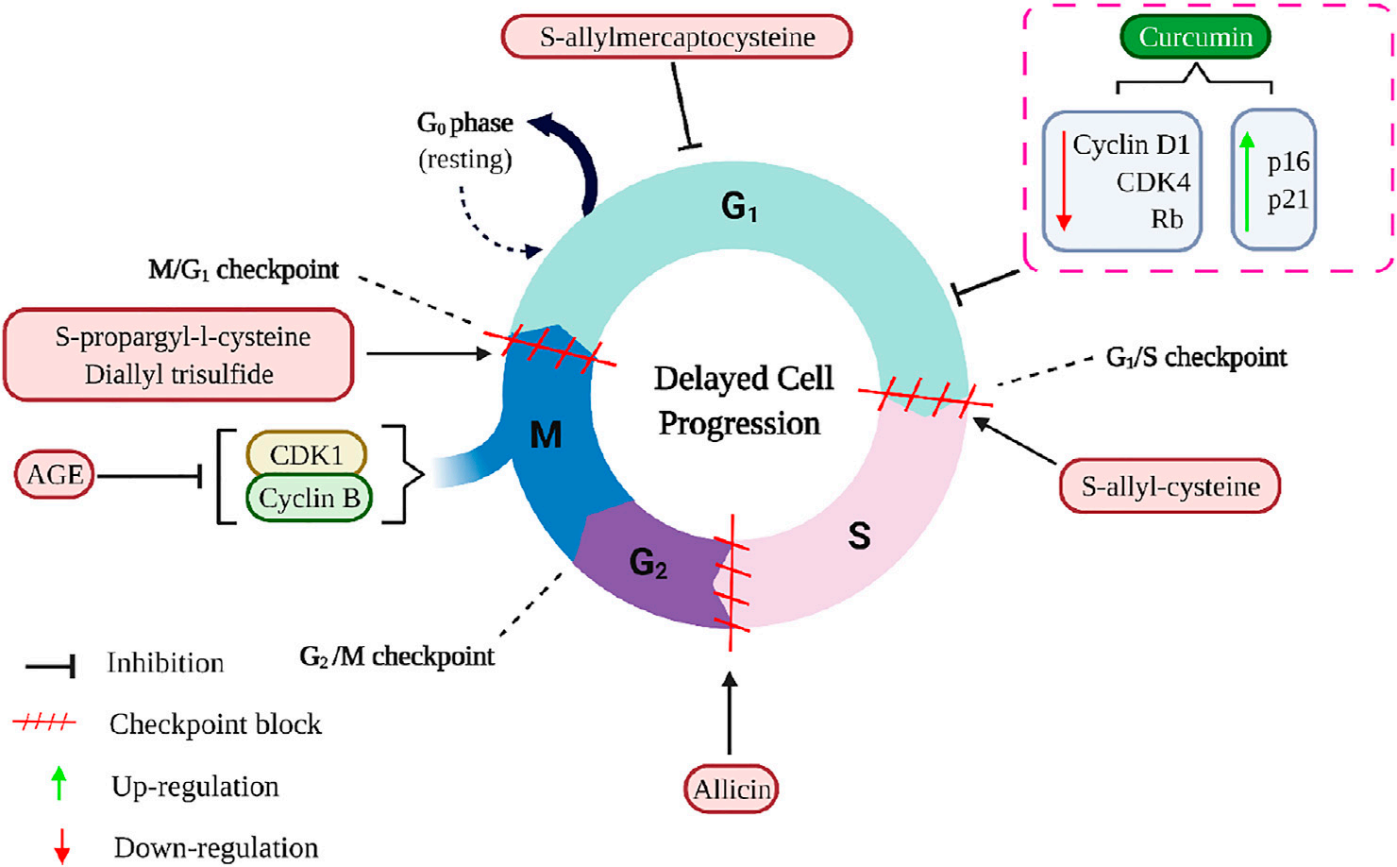
The mechanisms of curcumin and aged garlic extracts, including its active compounds, inhibit the cell cycle in cancer cells. AGE down-regulates Cyclin B1 and CDK1 and arrest the cell cycle in the G2/M- checkpoint. S-propargyl-L-cysteine and diallyl trisulfide arrest the cell cycle in the G2/M- checkpoint, whereas S-allyl-cysteine induced cell cycle arrest in the G1/S- checkpoint, and allicin induced cell cycle arrest during the S phase. Additionally, S-allylmercaptocysteine shortens the duration of the S phase and lengthens the duration of the G0/G1 phase. The dotted rectangle indicates the curcumin (lead compound of turmeric) functions. AGE, Aged garlic extract; CDK1, Cyclin-dependent kinase 1; CDK4, Cyclin-dependent kinase 4; Rb, Rb Protein.

Calebin A suppressed the expression of Nuclear Factor-kappa B (NF-κB), which promotes the anti-apoptotic B cell lymphoma extra-large (BCL-xL), B-cell lymphoma (BCL-2), surviving, proliferation (Cyclin D1), invasion (MMP-9), metastasis (CXCR4) biomarkers, as well as down-regulated apoptosis (Caspase-3) gene biomarkers, ultimately leading to apoptosis in human colorectal adenocarcinoma (HCT116) cells (Buhrmann et al., 2020). Rajitha et al. (Rajitha et al. (2016) demonstrated that the administration of 25 µM curcumin, a potential NF-κB inhibitor, significantly suppressed NF-κB activation *via* inhibiting the transcription factor E2F-1 and thymidylate synthase as compared to untreated cell lines. This treatment resulted in cell cycle arrest at the G0/G1 phase with a concomitant decrease in the number of cells in the S, and tumour growth was significantly reduced in the CRC cell lines HCT116 and HT-29. The western blotting analysis further revealed that curcumin significantly decreased the levels of cyclin D1, CDK4, and pRb and increased p16 and p21 in both cell lines compared to controls (Figure 8) (Rajitha et al., 2016). It has also been shown to emerge with anti-inflammatory and anti-tumour properties by the induction of apoptosis and modulating different signalling pathways, such as mitogen-activated protein kinase (MAPK), extracellular signal-regulated kinase (ERK), p38, Jun N-terminal kinase (JNK) in gastric cancer, and neurofibroma (Li et al., 2008; Lee et al., 2019). Curcumin and its analogues have been an effective chemotherapeutic agent and chemosensitizer by regulating specific microRNAs, signalling pathways, and epithelial-mesenchymal transition (Cai et al., 2018).

### 4.3 Garlic

Another commonly consumed spice is garlic (*Allium sativum* L.), a member of the family Liliaceae (Shang et al., 2019). Garlic can be consumed raw or cooked and in powder or oil form. Garlic as traditional medicine has been documented in ancient writings of Egypt, Greece, China, and India as early as 3,000 years ago (Rivlin, 2001; Omar and Al-Wabel, 2010). Garlic has been shown to reduce the incidence of heart disease and cancer in epidemiologic and preclinical investigations, and it has also been claimed to be an anti-cancer dietary component (Bayan et al., 2014). There are around 33 sulfur compounds, including alliin, allicin, ajoene, allyl propyl disulfide, diallyl trisulfide, S-allyl cysteine, vinyldithiines, S-allyl mercapto cysteine, and others, several enzymes (i.e., allinase, peroxidases, myrosinase), 17 amino acids like arginine and others, and minerals, such as selenium, germanium, tellurium and other trace minerals (Newall et al., 1996; Martins et al., 2016; Abe et al., 2020). As of 15 August 2021 (Scopus database), 19,339 papers related to the merits of garlic consumption have been documented between 1854 and 2021. One-third of these published records were related to its benefits in cancer modulation, while 8 % pivoted on its benefits for CRC prevention and management (Figure 1). There is compelling evidence that garlic and related sulfur components can reduce cancer risk and affect the biological behaviour of tumours. A high intake of garlic is associated with decreased risks for stomach and CRC (Omar and Al-Wabel, 2010).

#### 4.3.1 Lead Compounds of Garlic

**FIGURE 9:**
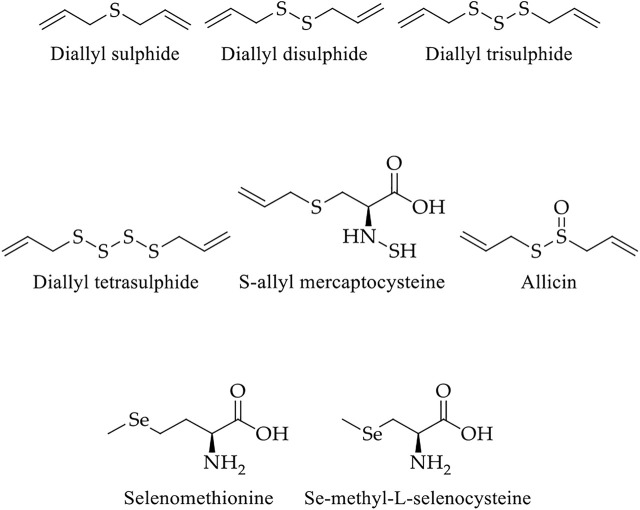
Chemical structure of main bioactive compounds of garlic.

Garlic’s pungent flavour renders it a daily seasoning or condiment in Asian cuisine. Prominent medicinal values and uses of garlic have been seen since ancient times. Garlic has more than 200 chemicals. The leading bioactive molecules among these compounds are diallyl sulfide, diallyl disulfide, diallyl trisulfide, diallyl tetrasulfide, S-allyl mercaptocysteine, allicin, selenomethionine, and se-methyl-L-selenocysteine (Figure 9) (Czepukojc et al., 2014). The potential chemotherapeutic activities of the aforementioned compounds in colorectal cell lines are listed in Figure 4 and Table 2.

#### 4.3.2 Anti-Colorectal Cancer Effects and Mechanisms of Actions of Garlic

Aged garlic extract (AGE) and its bioactive compounds are good chemopreventive agents for CRC because of their antiproliferative action on colorectal carcinoma cells and inhibitory activity on angiogenesis. AGE suppressed the proliferation of different CRC cell lines, namely DLD1, COLO205, HT29, SW480, and SW620 (Matsuura et al., 2006; Jikihara et al., 2015; Tung et al., 2015). It had diverse impacts on the invasive activities of these cell lines, and AGE showed a significant reduction in invasive activity on SW480 and SW620 cells; however, it did not affect the invasive activity of HT29 cells (Matsuura et al., 2006). There appears to be a relationship between the effect of AGE and the type of cancer cells being treated. AGE improved the endothelial cells’ adherence to collagen and fibronectin, whereas its bioactive compounds reduced cell motility and invasion. In addition, AGE had a strong inhibitory effect on the proliferation and tube formation of endothelial cells (Matsuura et al., 2006). SW620 is a metastasized SW480, and both cell lines have been documented to have increased p53 levels, while HT-29 cells consist of mutated p53 (Eng et al., 2021). Thus, it is plausible that the bioactive compounds interact with the molecules such as p53 that ensure cell cycle checkpoints are conducted rigorously.

AGE decreased the number of ACF but did not affect gross tumour pathology in the DLD1 human CRC cell line. AGE inhibited the proliferation of adenoma and adenocarcinoma lesions but did not affect normal colon mucosa. It delayed cell cycle progression by inhibiting cyclin B1 and Cyclin-dependent kinase 1 (cdk1) expression but did not trigger apoptosis in DLD1 (Jikihara et al., 2015). Bioactive compounds of garlic, particularly selenomethionine and se-methyl-L-selenocysteine, decreased ACF and induced apoptosis by about 80% by activating caspase 3. In addition, AGE delayed cell cycle progression by inactivation of NF-κB signalling and downregulation of cyclin B1 and cdk1 expression during the G2/M-phase (Figure 8) (Jikihara et al., 2015; Tung et al., 2015). Se-methyl-L-selenocysteine increased Fas and FasL expression, followed by the caspase-3, caspase-8, DNA fragmentation factor, and poly(ADP-ribose) polymerase cleavage. Se-methyl-L-selenocysteine also increased Bax protein levels while decreasing Bid and BCL-2 protein levels. However, this compound caused apoptosis *via* endoplasmic reticulum stress rather than reactive oxygen species stress. The cleavage of caspase-12 and caspase-9 increases growth arrest and protein levels of GADD 153 and 45. In COLO 205 cells, Se-methyl-L-selenocysteine reduced ERK1/2 and PI3K/AKT protein levels while increasing p38 and JNK protein levels (Tung et al., 2015).

These results suggested that AGE or garlic’s bioactive compounds, mainly selenomethionine and se-methyl-L-selenocysteine, could prevent tumour formation by inhibiting angiogenesis by suppressing endothelial cells motility, proliferation, and tube formation. Therefore, they could be good chemopreventive agents for CRC because of their antiproliferative action on colorectal carcinoma cells and inhibitory activity on angiogenesis.

### 4.4 Fenugreek

**FIGURE 10:**
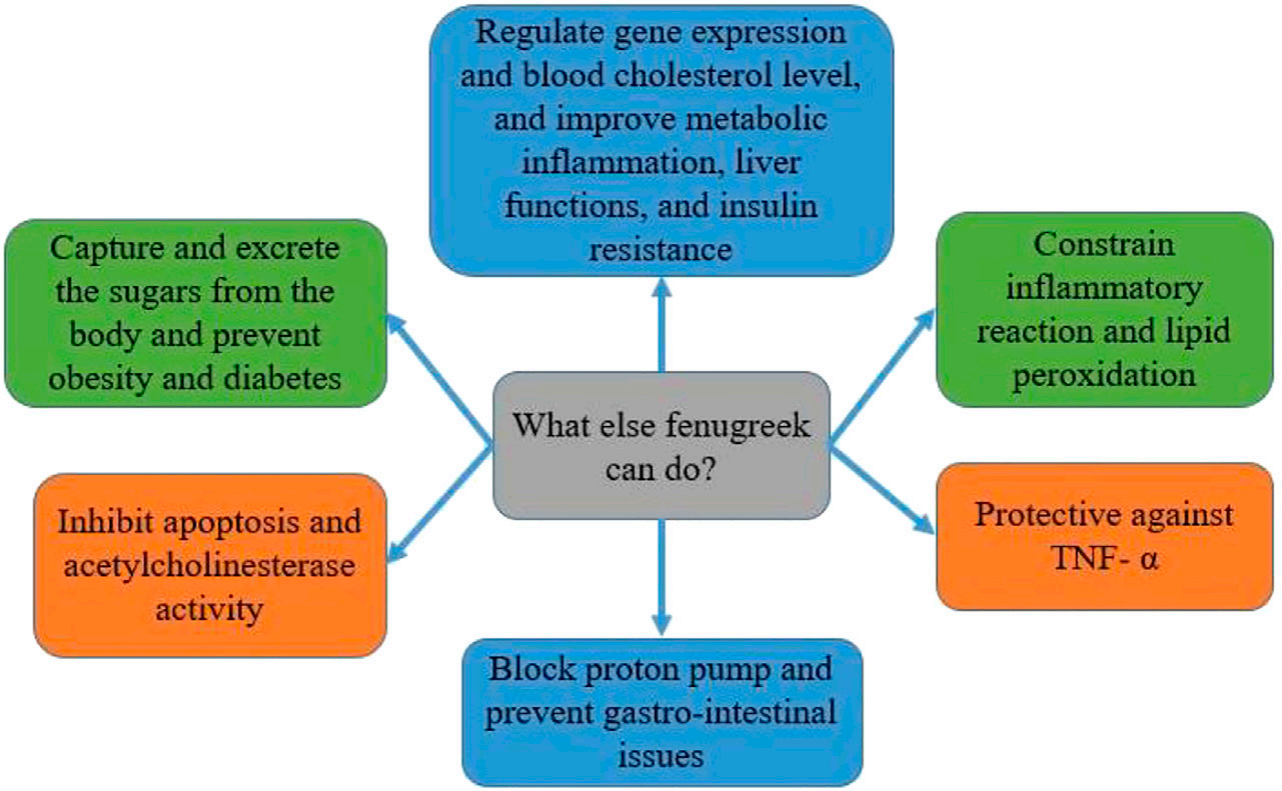
Health benefits and anticancer properties of fenugreek.

Fenugreek (*Trigonella foenum-graecum* L.) belongs to the family Leguminosae. It has long been used as a spice to improve the sensory quality of cuisines across the world, including in Bangladesh, India, and Pakistan (Wani and Kumar, 2018; Singh et al., 2020). Its seeds and green leaves, commonly used as leafy vegetables and seasonings, are now widely cultivated for medicinal purposes. They also enhance flavour, colour, and texture in foods (Tewari et al., 2020). Although modern medicine has made incredible advances, the usage of herbal plants for treating or preventing diseases is still widely used due to their diverse nutraceutical capabilities and safety. Among many spice crop plants that are nutritious, functional, and therapeutic, fenugreek is popular with all these characteristics. Recently, it has gained tremendous scientific attention for further evaluation and validation of nutraceutical and health benefits, especially lifestyle-related diseases and cancer. Our systematic investigation in the Scopus database revealed that scientists published around 3,713 papers between 1931 and 2021. Almost a quarter of the articles focused on cancers, and 5% of papers precisely focused only on CRC (Figure 1). The health benefits of fenugreek that lead to anti-cancer effects are summarised in Figure 10.

#### 4.4.1 Lead Compounds of Fenugreek

**FIGURE 11:**
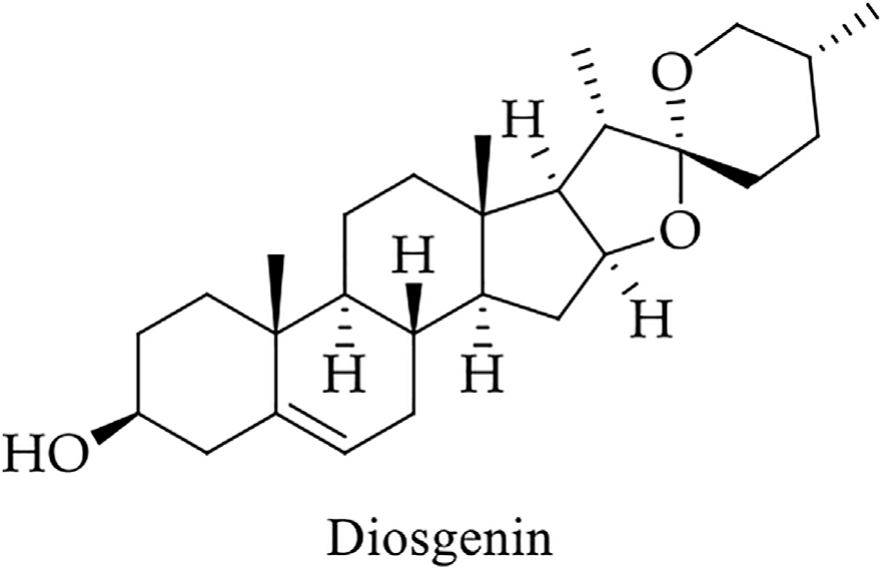
Chemical structure of the chemopreventive bioactive compound of fenugreek.

Since antiquity, fenugreek has been a member of the Fabaceae family and has been extensively utilized as Ayurveda in traditional and alternative medicine systems (Aasim et al., 2018; Rajasekaran, 2019). It is rich in several phytochemicals, of which diosgenin (a saponin) (Figure 11) has anticarcinogenic properties (Table 2) (Raju et al., 2004).

#### 4.4.2 Anti-Colorectal Cancer Effects and Mechanisms of Actions of Fenugreek

Multiple functional and molecular targets are involved in the anti-cancer effects of fenugreek or its bioactive compounds, such as apoptosis in tumour cell lines, especially in human CRC (Table 2) (Raju et al., 2004). In 1,2-Dimethylhydrazine-treated mice, a diet rich in fenugreek seed powder reduced colon tumour incidence and lipid peroxidation LPO while simultaneously increasing GPx, glutathione S-transferase (GST), SOD, and catalase activity in the liver (Sushma and Devasena, 2010). Another study demonstrated that diosgenin inhibited the production of azoxymethane (AOM)-induced aberrant crypt foci, a preneoplastic colonic lesion in F344 rats. This compound induced apoptosis in HT-29 human colon cancer cells by suppressing BCL-2 and activating caspase-3 protein expression, implying its potential as a colon cancer preventive agent (Raju et al., 2004).

### 4.5 Sesame

Sesame (*Sesamum indicum*) from the Pedaliaceae family is one of the earliest domesticated oilseed crops known to humankind with its multifarious uses. It is mainly consumed in various cuisines and preferably used with bread, biscuits, crackers, and so forth and as a seasoning in food worldwide (Namiki, 2007). Sesame has an essential role in human nutrition due to its rich chemical compositions like oil (44–58%), protein (18–25%), carbohydrates (∼13.5%), minerals and vitamins (Elleuch et al., 2007; Hassan, 2012; Lim, 2012). Sesame seeds have multiple potential bioactive compounds that are beneficial components in food and are accountable for disease-preventing properties. These chemical compounds include phenolics, carotenoids, phytosterols, and polyunsaturated fatty acids, often utilized as antioxidants and for other purposes (Pathak et al., 2017). Recent studies demonstrated that the leaves and shoots of sesame plants are used as vegetables, and the leaves contain valuable nutrients such as amino acids responsible for various traditional uses, including pain relief, catarrh, eye pain, bruises, and erupted skin lesions. In Japan, young sesame leaves (30–70 cm tall, 40–60 days after planting) are dried and sold as a health food supplement (Fuji et al., 2018).

The seeds contain lignans such as sesamin and sesaminol and are highly valued as traditional health and nutraceutical food. Young sesame leaves contain three iridoids (lamalbid, sesamoside and shanzhiside methyl ester) and seven polyphenols (cistanoside F, chlorogenic acid, pedalitin-6-O-laminaribioside, pedaliin, isoacteoside, pedalitin and martynoside), and acteoside. These compounds show potential radical scavenging effects in assays like the DPPH, ABTS, and superoxide anion radicals test (Matsufuji et al., 2011; Fuji et al., 2018). However, sesamin, a major lignan in sesame oil did not show antioxidant *in vitro* activity (Nakai et al., 2003). Interestingly, on the other hand, this compound showed protective effects against oxidative damage in rat liver. The same metabolites were found as glucuronic acid and/or sulfuric acid conjugates in substantial amounts in rat bile after oral administration of sesamin (Nakai et al., 2003). Therefore, sesamin is considered as a prodrug. On giving 10 mg/kg or 100 mg/kg of sesamin (S10, S100) to 32 male ddY mice 2 h before swimming exercise using a new forced-swimming apparatus, their plasma lipid peroxide level was significantly suppressed, while the level in the control group increased significantly after exercise (*p* < 0.01). S100 showed significantly higher total GPx activity and GST activity in the liver compared to control (*p* < 0.05) (Ikeda et al., 2003). This finding suggested that sesamin may enhance liver LPO degradation, resulting in strong protective effects against exercise-induced plasma lipid peroxidation. Sesamin *in vivo* metabolites with the catechol group is the most efficient antioxidants (Papadopoulos et al., 2016). The highly antioxidative action of sesame oil has been clarified, and it has been determined that recently discovered lignans mediate with tocopherols. A novel synergistic effect of sesame lignans with tocopherols has been found, and it is believed to be responsible for the antiaging effect of sesame. Sesame lignans inhibit metabolic decomposition of tocopherols, which results in the antiaging effect of sesame being attributed to strong vitamin E activity (Namiki, 2007). A systematic search in the Scopus database showed that around 11,089 articles were published between 1898 and 2021. Among these publications, about 15% emphasized cancers, and 2% precisely concentrated on CRC alone (Figure 1).

#### 4.5.1 Lead Compounds of Sesame

**FIGURE 12:**
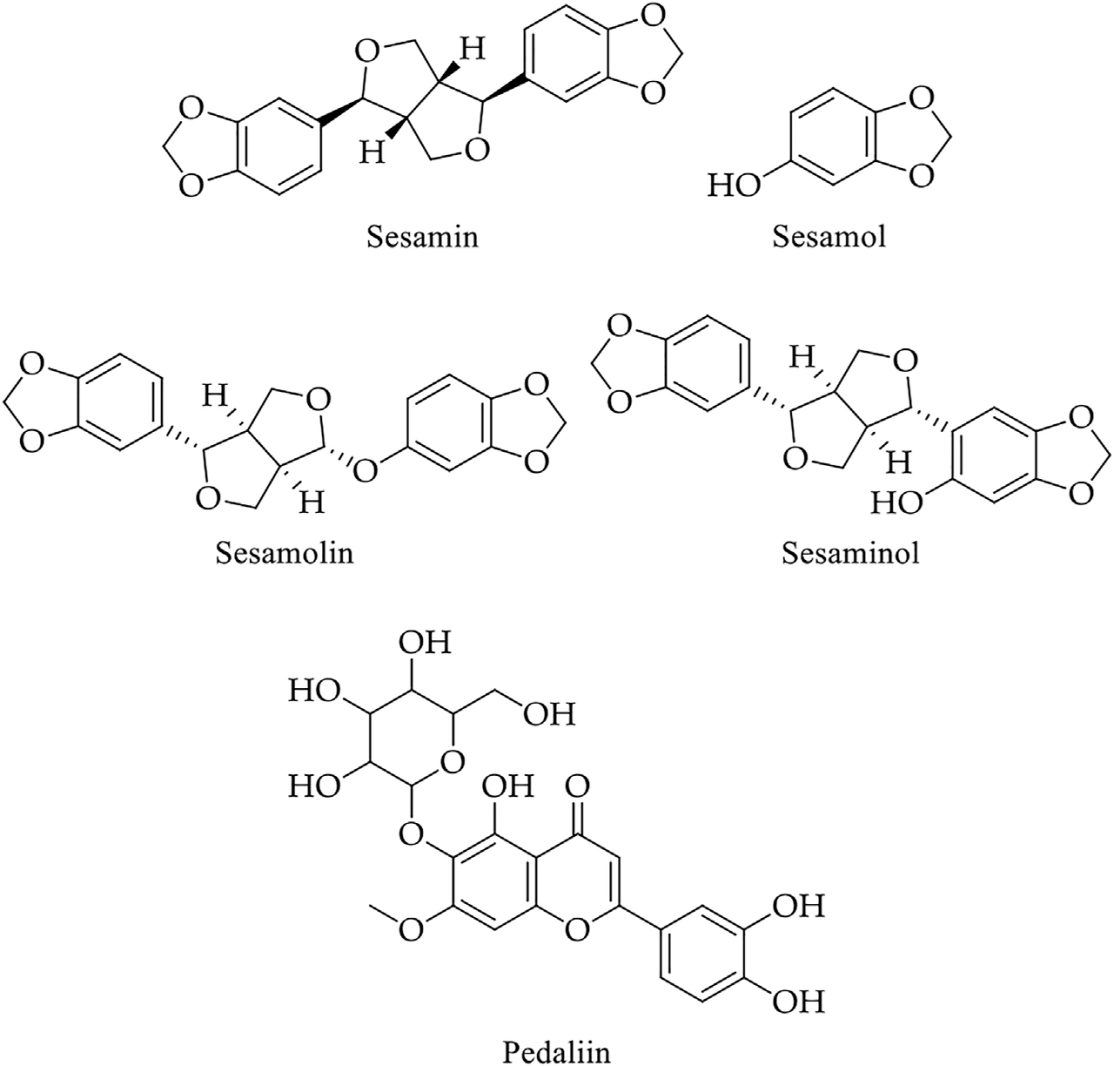
Chemical structure of main bioactive compounds of sesame.

Sesame from the Pedaliaceae family generally refers to sesame seeds. It is one of the oldest condiments and a commercially significant oilseed crop (Queen of oilseed crops) with 40–60% oil and medicinal value due to the presence of a broad spectrum of bioactive molecules, including sesamin, sesamol, sesamolin, and sesaminol (Figure 12 and Table 2) (Zohary and Hopf, 2000; Aasim et al., 2018).

#### 4.5.2 Anti-Colorectal Cancer Effects and Mechanisms of Actions of Sesame

Sesamol is one of the prominent biomolecules of sesame seeds that confers chemopreventive properties and analgesic effects (Table 2). It targets p53, MAPK, JNK, PI3K/AKT, TNF, NF-B, PPAR, caspase-3, Nrf2, eNOS, and LOX signalling pathways, suggesting that sesamol possesses potent anti-cancer properties. It has a wide range of biological functions, including inhibition of lipid peroxidation and enhancement of radical scavenging, upregulation of antioxidant enzymes, suppression of TNFα and IL-1β expression, inhibition of NF-κB signalling, suppression of LOX-1 and 5-LOX activity, induction of apoptosis, arresting cell growth at different phases of the cell cycle and modulation of caspase-3, p53, BAX, and BCL-2 expression (Gupta et al., 2009; Chu et al., 2010a; Chu et al., 2010b; Wu et al., 2015; Kim et al., 2021). In HCT116 cells, sesame leaf extract (250 g/ml and 500 g/ml) induced apoptosis and cell cycle arrest during the G2/M phase. This extract increased the G2/M cell population to 2.3–6.6-fold of the control, with a concurrent drop in G0/G1 and S phase cell populations, demonstrating that sesame leaf extract has a G2/M arresting function (Kim et al., 2021).

### 4.6 Flaxseed

Flaxseed (*Linum usitatissimum* L.) from the Linaceae family is one of the world’s oldest cultivated herbaceous crops. It is still widely grown for its oil, fibre, and nutritional value. Flaxseed oil is high in omega-3 fatty acid linolenic acid (55%), an attribute that boosts its role as a functional food. Additionally, flaxseeds are used in animal feed to boost reproductive health (Oomah, 2001; Turner et al., 2014). The flaxseed products include whole seed (ground), flaxseed oil (partially defatted), fully defatted (solvent extraction), mucilage extract, flaxseed hull, oleosomes, and alcohol extract. Each of these products has particular health benefits. Reports typically neglect the presence of many bioactive chemicals in flaxseed fractions or attribute the impact to a single component. However, whole flaxseed is widely accepted as a healthy food with anti-cancer activity (Shim et al., 2014). In female rat mammary glands, flaxseed flour reduces epithelial cell proliferation and nuclear abnormalities that indicate the reduction of mammary tumour growth in the later stages of carcinogenesis (Serraino and Thompson, 1991; Thompson et al., 1996). Recently the growth of cancer research using flaxseeds has increased significantly. Our team’s systematic literature search (Scopus database) revealed that about 7,357 articles were published between 1844 and 2021. About 22% concentrated on cancers among these publications, and 4% was specifically spotlighted on CRC (Figure 1).

#### 4.6.1 Lead Compounds of Flaxseed

**FIGURE 13:**
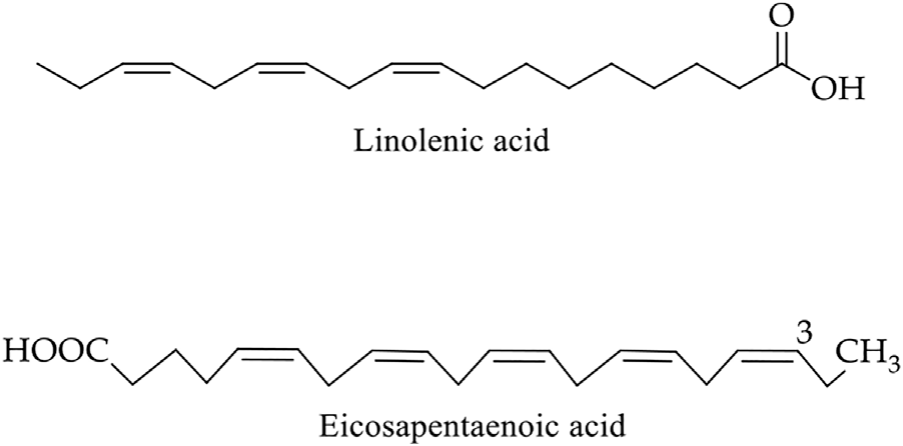
Chemical structure of main bioactive compounds of flaxseed. α-Linolenic acid (18:3n-3) and eicosapentaenoic acid (20:5n-3) belong to the omega-3 fatty acids group.

Flaxseed is one of the world’s oldest crops, grown since the dawn of civilization. Flaxseed treated various ailments in India, Sri Lanka, Greece, Rome, Egypt, and many other countries and enriched the Ayurveda and traditional Chinese medicine system (Goyal et al., 2014). Due to its high fibre level, omega-3 fatty acids, flavonoids, and phytoestrogens, flaxseed usage in reducing human CRC risk are gaining attention (Calviello et al., 2007; Lattimer and Haub, 2010; Kajla et al., 2015). Moreover, flaxseed is one of the most significant plant sources of linolenic acid, an omega-3 polyunsaturated fatty acid (PUFA) (49–60%), which is linked to a lower risk of colonic neoplasms (Figure 13 and Table 2) (Goyal et al., 2014; DeLuca et al., 2018).

#### 4.6.2 Anti-Colorectal Cancer Effects and Mechanisms of Actions of Flaxseed

*In vitro* studies and animal models suggest that diets high in 3-PUFA may protect against malignancies, such as colon cancer, whereas treatment with 6-PUFA may inhibit cancer cells proliferation. For example, mice fed diets enriched in α-linolenic acid, which enhanced plasma levels of α-linolenic acid (ALA) and its metabolites eicosatetraenoic acid (EPA) and docosahexaenoic acid (DHA), showed a reduction in the growth of transplanted colon cancer cells (Danbara et al., 2005; Bommareddy et al., 2010; Chamberland and Moon, 2015). In case-2 human colon adenocarcinoma cells, ALA has been demonstrated to inhibit cell proliferation and induce apoptosis (Bommareddy et al., 2010). SW480 cells treated with enterolignans (enterodiol (ED) alone or in combination with enterolactone (EL)) showed a dose-dependent reduction in cell number, induction of S-phase cell cycle arrest, elevated cyclin A protein levels, an increased proportion of apoptotic cells (0–40 mol/L). Similar results were observed in a study using Colo 201 cells treated with EL, where apoptosis was modulated. Cell proliferation was decreased by the up-regulation of an apoptosis-inducing protein (a cleaved form of Caspase-3) and the down-regulation of both an apoptosis-inhibiting protein (BCL-2) and proliferation cell nuclear antigen (PCNA) protein (Danbara et al., 2005). Regulation of transcription in apoptotic genes (BCL-2, CCND1, and c-Myb) and cell cycle regulation were reported using Young Adult Mouse Colonocytes treated with low levels of EL (1 M) and ED (5 M) (DeLuca et al., 2018).

## 5 The Way Forward

Significant ground-breaking knowledge of molecular mechanisms behind CRC development revealed that dietary factors might be associated with CRC development at an increasing rate. However, the evidence to date is regrettably inadequate due to its highly complex mechanisms. Another significant association of intestinal microbiota with CRC has been predicted. Again, intestinal microbiota balance depends on the dietary habits and alterations of balanced intestinal microbiota involved with CRC development and progression (Leeming et al., 2019; Wong and Yu, 2019). However, modulation of the gut microbiota is a promising strategy to enhance treatment efficacy and reduce the adverse effects of CRC therapies (Wong and Yu, 2019). Many challenging issues, including aetiology, diagnosis, treatment, and management, need to be addressed to properly manage CRC and identify the key concerns for a long-term solution.

For appropriate management of CRC, several steps are required to follow for this global issue. Besides early identification and screening of high-risk communities and individuals, taking preventive measurements through consuming high dietary foods and maintaining a normal lifestyle is essential. This study revealed that consuming culinary herbs and spices might help prevent and cure CRC. They showed potential growth inhibition of human colorectal cancer cells by regulating relevant molecular signalling pathways. The bioactive compounds from these herbs and spices can be isolated and purified through several techniques, including convention extraction or green techniques like supercritical or subcritical fluid extraction. Some sophisticated analytical tools can be applied to purify and identify pure compounds, such as the High-Pressure Liquid Chromatography (HPLC) technique, Liquid chromatography-mass spectrometry (LC-MS) analysis, LC-MS-mass spectrometry (LC-MS-MS), and Gas chromatography-mass spectrometry (GC-MS) (Hossain et al., 2014; Hossain S. et al., 2021). To develop stable drugs, isolated compounds can be formulated in different drug forms like tablets, suspension or emulsion (Hua, 2019). They also might be incorporated with nanoparticles and encapsulated in the biodegradable polymer for target-specific drug delivery (Begines et al., 2020).

Some additional measurements are highly recommended as follows: 1) public cancer registration for tracking CRC incidence and survival, 2) government should provide quality medical care for timely diagnosis and treatment, 3) ensure better-personalized therapy and easy access to clinical trials for CRC patients, and 4) increased awareness of CRC as well as about other comorbidities to improving cancer care and research for proper management of this global issue.

## 6 Conclusion

Literature has provided evidence that herbs and spices have potential roles in preventing and reducing CRC severity. All the six common herbs and spices, namely ginger, turmeric, garlic, fenugreek, sesame, and flaxseed, are useful in preventing CRC. Apart from chemotherapeutic uses, these culinary herbs and spices-derived substances could have a salubrious indication for CRC prevention and management. Their mechanisms of action are mainly mediated through BCL-2, K-ras, and MMP pathways, caspase activation, the extrinsic apoptotic pathway, and the regulation of ER-stress-induced apoptosis. Therefore, these herbs and spices are good candidates for chemopreventive agents for CRC due to their antiproliferative action on colorectal carcinoma cells and inhibitory activity on angiogenesis.
