# Supplementary material for: Herb and Spices in Colorectal Cancer Prevention and Treatment: A Narrative Review
Source: Front Pharmacol. 2022 Jun 30;13:865801. doi: 10.3389/fphar.2022.865801 (PMC9280164; doi:10.3389/fphar.2022.865801)
Supplement: Supplementary file 1 [file Table1.DOCX]

Supplementary Material

# Supplementary Table

**Table S1:** FDA Approved Drugs for Colorectal Cancer [1].

| No. | Drugs |
| --- | --- |
|  | Avastin (Bevacizumab) |
|  | Camptosar (Irinotecan Hydrochloride) |
|  | Capecitabine |
|  | Cetuximab |
|  | Cyramza (Ramucirumab) |
|  | Eloxatin (Oxaliplatin) |
|  | Erbitux (Cetuximab) |
|  | 5-FU (Fluorouracil Injection) |
|  | Fluorouracil Injection |
|  | Ipilimumab |
|  | Irinotecan Hydrochloride |
|  | Keytruda (Pembrolizumab) |
|  | Leucovorin Calcium |
|  | Lonsurf (Trifluridine and Tipiracil Hydrochloride) |
|  | Mvasi (Bevacizumab) |
|  | Nivolumab |
|  | Opdivo (Nivolumab) |
|  | Oxaliplatin |
|  | Panitumumab |
|  | Pembrolizumab |
|  | Ramucirumab |
|  | Regorafenib |
|  | Stivarga (Regorafenib) |
|  | Trifluridine and Tipiracil Hydrochloride |
|  | Vectibix (Panitumumab) |
|  | Xeloda (Capecitabine) |
|  | Yervoy (Ipilimumab) |
|  | Zaltrap (Ziv-Aflibercept) |
|  | Zirabev (Bevacizumab) |
|  | Ziv-Aflibercept |

**Table S2.** FDA Approved Drugs Combination for Colorectal Cancer [1].

| No. | Combination | Acronym | Characteristics |
| --- | --- | --- | --- |
|  | CAPOX | CAP= Capecitabine  OX= Oxaliplatin | The advanced stage that starts to spread nearby tissue, lymph nodes, or distant parts of the body |
|  | FOLFIRI | FOL= Leucovorin Calcium (Folinic Acid)  F= Fluorouracil  IRI= Irinotecan Hydrochloride | - |
|  | FOLFIRI- BEVACIZUMAB | FOL= Leucovorin Calcium (Folinic Acid)  F= Fluorouracil  IRI= Irinotecan Hydrochloride  + Bevacizumab | Has metastasized (spread to other parts of the body) |
|  | FOLFIRI- CETUXIMAB | FOL= Leucovorin Calcium (Folinic Acid)  F= Fluorouracil  IRI= Irinotecan Hydrochloride  + Cetuximab | Has certain mutation of the *KRAS* gene and has metastasized (spread to other parts of the body) |
|  | FOLFOX | FOL= Leucovorin Calcium (Folinic Acid)  F= Fluorouracil  OX= Oxaliplatin | - |
|  | FU-LV | FU= Fluorouracil  LV= Leucovorin Calcium | - |
|  | XELIRI | XEL= Capecitabine (Xeloda)  IRI= Irinotecan Hydrochloride | Has metastasized (spread to other parts of the body) |
|  | XELOX | XEL= Capecitabine (Xeloda)  OX= Oxaliplatin | Advance stage that started to spread nearby tissue, lymph nodes, or distant parts of the body |

**Reference**

1. PDQ Colon Cancer Treatment. Available online: <https://www.cancer.gov/types/colorectal/hp/colon-treatment-pdq> (accessed on 15 July 2021).
